# ATP-P2X_7_R-mediated microglia senescence aggravates retinal ganglion cell injury in chronic ocular hypertension

**DOI:** 10.1186/s12974-023-02855-1

**Published:** 2023-07-31

**Authors:** Miao Wei, Guowei Zhang, Zeyu Huang, Xuemeng Ding, Qing Sun, Yujian Zhang, Rongrong Zhu, Huaijin Guan, Min Ji

**Affiliations:** 1 Eye Institute, Affiliated Hospital of Nantong University, Medical School of Nantong University, 20 Xisi Road, Nantong, 226001 China; 2 Dalian Medical University, Dalian, 116000 China

**Keywords:** Glaucoma, Microglial senescence, P2X_7_R, Mitophagy, Bone marrow cell transplantation

## Abstract

**Background:**

Dysfunction of microglia during aging affects normal neuronal function and results in the occurrence of neurodegenerative diseases. Retinal microglial senescence attributes to retinal ganglion cell (RGC) death in glaucoma. This study aims to examine the role of ATP-P2X_7_R in the mediation of microglia senescence and glaucoma progression.

**Methods:**

Forty-eight participants were enrolled, including 24 patients with primary open-angle glaucoma (POAG) and age-related cataract (ARC) and 24 patients with ARC only. We used ARC as the inclusion criteria because of the availability of aqueous humor (AH) before phacoemulsification. AH was collected and the adenosine triphosphate (ATP) concentration was measured by ATP Assay Kit. The chronic ocular hypertension (COH) mouse model was established by microbead occlusion. Microglia were ablated by feeding PLX5622 orally. Mouse bone marrow cells (BMCs) were prepared and infused into mice through the tail vein for the restoration of microglia function. Western blotting, qPCR and ELISA were performed to analyze protein and mRNA expression in the ocular tissue, respectively. Microglial phenotype and RGC survival were assessed by immunofluorescence. The mitochondrial membrane potential was measured using a JC-1 assay kit by flow cytometry.

**Results:**

ATP concentrations in the AH were increased in older adults and patients with POAG. The expression of P2X_7_R was upregulated in the retinal tissues of mice with glaucoma, and functional enrichment analysis showed that P2X_7_R was closely related to cell aging. Through in vivo and in vitro approaches, we showed that pathological activation of ATP-P2X_7_R induced accelerated microglial senescence through impairing PTEN-induced kinase 1 (PINK1)-mediated mitophagy, which led to RGC damage. Additionally, we found that replacement of senescent microglia in COH model of old mice with BMCs from young mice reversed RGC damage.

**Conclusion:**

ATP-P2X_7_R induces microglia senescence by inhibiting PINK1-mediated mitophagy pathway. Specific inhibition of ATP-P2X_7_R may be a fundamental approach for targeted therapy of RGC injury in microglial aging-related glaucoma.

**Supplementary Information:**

The online version contains supplementary material available at 10.1186/s12974-023-02855-1.

## Introduction

The development of primary glaucoma is highly age-related, with an increased prevalence in people over 50 years of age. The disease is characterized by the death of retinal ganglion cells (RGC) [1, 2]. Recent studies have demonstrated that microglia senescence aggravates the risk of neurodegenerative diseases [3], and reversing aging microglia can effectively protect neuronal function and prevent the occurrence of neurodegenerative diseases such as cognitive impairment in aged individuals [4]. The neural protective function of macroglia decreases with age, which strides toward retinal neurodegeneration, including primary glaucoma [5]. However, the molecular mechanism of retinal microglial senescence and its role in glaucoma RGC injury remains unclear.

Intracellular adenosine triphosphate (ATP) is a major energy metabolite, while extracellular ATP is an essential inflammatory mediator, neurotransmitter, and glial cell transmitter, which mediates various cellular interactions [6]. Extracellular ATP increased in senescent nervous tissues [7]. Purine receptor P2X_7_R, a natural ligand of ATP and 3′-O-(4-benzoylbenzoyl) ATP (BzATP), is mainly expressed in immune cells [8]. Under physiological conditions, the activation of P2X_7_R regulates the differentiation and maturation of microglia, while pathological activation of P2X_7_R amplifies the inflammatory response and induces tissue damage [9]. P2X_7_R mediates cell phagocytosis in the resting state. Once P2X_7_R is activated, the phagocytic function of microglia is inhibited [9]. Hence, exploring the mechanism of ATP-P2X_7_R in retinal microglia under pathological conditions may be the key to clarifying glaucoma progression and identifying targeted therapies.

Cell senescence is a permanent state of cell cycle arrest caused by different stimuli, accompanied by a series of pathological changes [10]. Many studies have reported that enhanced mitophagy can ameliorate cell aging [11, 12]. Mitophagy is a specialized form of autophagy that removes aged and damaged mitochondria. Mitophagy promotes the repair and regeneration of mitochondria, which is especially important for long-lived cells such as microglia [13]. Mitophagy also exerts physiological functions, such as promoting cell differentiation and delaying aging. Dysregulation or impairing its regulatory mechanisms leads to physiological aging and age-related diseases [14, 15]. However, the mechanism of mitophagy in retinal microglial senescence is still unknown.

In this study, we explored the mechanism of microglial senescence induced by ATP-P2X_7_R activation and explored the role of reversing microglial senescence in RGC damage in vitro and in vivo. The present study would provide the scientific basis for potential drugs to block microglial senescence and lay a foundation for microglia transplantation to treat RGC injury in glaucoma.

## Materials and methods

### Study participants

Forty-eight participants were enrolled in this study at the Department of Ophthalmology, Affiliated Hospital of Nantong University, including 24 patients with primary open-angle glaucoma (POAG)/age-related cataract (ARC) and 24 patients with ARC only whose lens opacity matched with that of the POAG group. We used ARC as the inclusion criteria because of the availability of aqueous humor (AH) before phacoemulsification. The participants were self-reported to be Han Chinese. All eyes that underwent complex cataract surgery, were diagnosed with POAG, and had no incisional surgery for 1 year before and 1 year after phacoemulsification were considered for inclusion. Eyes were subsequently excluded if they had a prior incisional glaucoma surgery, if phacoemulsification was combined with another surgery was not confirmed from the operative report. Furthermore, patients in the POAG group were excluded if they did not have a confirmed glaucoma diagnosis or if they had non-POAG types of glaucoma such as neovascular, uveitis, or chronic angle closure. The control group was made up of patients without POAG, who underwent phacoemulsification with intraoperative mechanical pupillary expansion. Control patients were excluded if they had incisional surgery 1 year before phacoemulsification. The subjects were further divided into two groups by age, e.g., < 65 years and > 75 years [16]. All procedures were performed with informed consent and approved by the Affiliated Hospital of Nantong University and followed the Declaration of Helsinki.

AH (0.1–0.2 ml) was collected from the 48 patients (48 eyes) from the central region of the pupil before the cataract surgery, and ATP concentration of AH was assessed using an Enhanced ATP Assay Kit (Beyotime, Shanghai, China) according to the manufacturer’s instructions.

### Bioinformatics analysis

We selected the GSE26299 dataset from the GEO database (https://www.oncomine). The GSE26299 data included 31 retinal tissues of non-glaucoma mice and 18 retinal tissues of moderate and severe glaucoma mice [1]. For further analysis, mice were divided into the P2X_7_R high and low expression groups, and the “limma” R package was used to analyze the differentially expressed genes (DEGs) with the conditions: |logFC|> 1 and P < 0.05. The DEGs were subjected to Gene Ontology (GO) via the DAVID 6.8 (https://david.ncifcrf.gov/). The GO terms are then visualized via R Package "ggplot2" with the conditions: FDR < 0.05 and counts ≥ 4.

### Animals and chronic ocular hypertension model (COH)

C57BL/6 mice were purchased from the Laboratory Animal Center of Nantong University. All animal protocols were followed by the National Institutes of Health (USA) Guidelines for the Use of Laboratory Animals.

The mouse COH model was established as described previously [2]. The mice were divided into young group (2 months old) and old group (20 months old) according to age. Mice were randomly assigned to four groups: (1) young sham group (n = 6); (2) young COH group (n = 4); (3) old sham group (n = 6); (4) old sham group (n = 4). After the mice were anesthetized by intraperitoneal injection of 40 mg/kg sodium pentobarbital, the eyeballs were dilated. The tip of a 1-ml syringe was used to make a corneal tunnel in the upper part of the center of the cornea. A 10-µl syringe connected to the tip of a capillary glass tube was used to inject a bubble into the anterior chamber, and then 2 µl microbeads (FluoSphere, Thermo Fisher, USA) were injected into the anterior chamber slowly. Reinjection was performed 2 weeks after the first injection to maintain high intraocular pressure (IOP). Aureomycin ointment was applied to both eyes of the animals after the operation to reduce the occurrence of postoperative infection. IOP was regularly measured with a TonoLab tono-pen tonometer, and the average of three measurements was recorded. The sample collection and end-point measurements were taken at -2 months and -20 months.

### Retrograde labeling and counting of ganglion cells

Seven days before the mice were killed, the mice were fixed on a stereotaxic frame after being anesthetized, and 1.5 µl 2% fluorogold (FG; Biotium, Hayward, CA, USA) was injected into two sites of the superior colliculus (5.9 and 6.4 mm posterior and 1.4 mm lateral to bregma and 4.0 mm deep). The mice were killed, and the eyes were fixed with 4% paraformaldehyde for 30 min before retinal staining. Images were captured using a fluorescence microscope, and the number of labeled cells in 12 photos per retina (3 photos per retinal quadrant) at 1/6, 3/6, and 5/6 of the retinal radius was summed. The data are expressed as the mean RGC density/mm^2^ for each group.

### Bone marrow cells transplantation

The obliteration of microglia was conducted by feeding PLX5622 orally (PLX5622-containing chow, Plexxikon, USA), 1200 mg daily for 14 days. PLX5622-treated old mice (n = 10) were served as the recipients of myeloid cells separated from the bone marrow of young mice. The 4- to 6-week-old GFP-C57BL/6J mice as much were served as the donor. Bone marrow cells (BMCs) were harvested under sterile conditions. A single-cell suspension was prepared at the cell density of 1 × 10^9^/ml and stored on ice until transplantation. After anesthesia, 10 μl of suspended donor BMC was injected into the tail veins of the recipient mice with a sterile 26 G needle.

### Contrast sensitivity

Spatial frequency thresholds were measured using the staircase paradigm. Step method was used to determine the spatial frequency from 0.15 to 0.40 cycles/degree. Rotation speed (12°/s) and contrast ratio (100%) remain constant. Their spatial frequency was systematically increased in successive trials until the animals ceased to track the moving stimulus.

### Microglia purification from adult mice retina

 ~ 2 and ~ 20-month-old C57BL/6 mice (n = 3 each group) were anesthetized with pentobarbital (40 mg/kg, intraperitoneal injection) and the eyeballs were removed under sterile conditions [16, 17]. The cornea and lens are removed and the retina is carefully removed from the eye cup under the microscope. Six retinal tissue were brought together by mechanical dissociation and digested at 37 °C for 30 min with gentle shaking in a buffer containing papain, dispase II, and DNase I followed. The mixed retinal single-cell suspension was washed in PBS and filtered through a 100-μm cell filter. Centrifuge cell suspension, 300 × *g*, 5 min, complete absorption of supernatant. Each 10^7^ cells were re-suspended in a 90 µL buffer. The cells were incubated with 10 μl Anti-CD11b magnetic microbeads (130–1093-634, Miltenyi Biotec) at 4 °C for 20 min, sheltered from light. The cells were washed with PBS and centrifuged at 300 × g for 5 min. The supernatant is aspirated and re-suspended in the PBS buffer. The cell suspension was applied to a column fixed in a magnetic separator (130-042-302, Miltenyi Biotec) using a 3 ml PBS pre-balanced LS column (130–042-401, Miltenyi Biotec). Wash the column with 10 ml PBS. The column was removed from the separator and the magnetically labeled cells were rinsed into a 15 ml collection tube with 5 ml PBS. The magnetically labeled cells were centrifuged at 300 × *g* for 5 min. The supernatant was aspirated, re-suspended in an appropriate buffer, planted on a 24-well plate coated with poly-l-lysine, and further phagocytosis experiment was performed [18].

### Cell culture and treatment

One-day-old mice were used to prepare the primary retinal microglia and primary RGC cultures according to our previously described procedures [3]. The cells were identified by immunocytochemical staining with specific antibodies (Additional file 2: Fig. S1). The number of positive cells in six random fields of each Petri dish was counted using an Olympus IX71 microscope. A microglial cell line BV2 was donated by the Department of Neuroscience, Nantong University. Microglia were cultured in high-glucose medium supplemented with 20% fetal bovine serum (Thermo Fisher Scientific), 100 IU/mL penicillin and 100 IU/mL streptomycin. The cell line algebra used is less than 10 generations. All cells were incubated at 37 °C in a humidified atmosphere containing 5% CO_2_. The cells at 70–80% confluence were treated with BzATP, A438079 and Carbonyl cyanide m-chlorophenylhydrazine (CCCP, Sigma-Aldrich, St. Louis, MO, USA). An equal volume of DMSO was utilized as control.

### Cell transfection

The PTEN-induced kinase 1 (PINK1) coding region was cloned into pcDNA3.1 vector. Cells were transfected with PINK1 overexpression and empty vector plasmids using Lipofectamine 2000 according to the manufacturer's instructions (Invitrogen, Carlsbad, CA). Twelve hours after transfection, the cells were harvested for western blotting and subsequent experiments.

### Cell β-galactosidase staining

The Cell aging β-galactosidase Staining Kit (Beyotime, Shanghai, China) was used for the detection of senescent cells or tissues since senescence-associated β-galactosidase (SA-β-Gal) activity is increased during aging. Microglia were seeded in 6-well plates and pretreated. The cells were washed three times with phosphate-buffered saline (PBS), 1 mL fixative solution was added to each well and the cells were incubated for 15 min at room temperature. After washing with PBS three times, the samples were mixed with staining solution and incubated overnight at 37 °C (THUNDER Imaging Systems, Leica).

### Western blotting

Protein expression was analyzed by Western blotting as described previously [4]. Briefly, protein lysates (20 µg) were separated by electrophoresis on sodium dodecyl sulfate–polyacrylamide gels and then transferred onto polyvinylidene fluoride membranes. After blocking with 4% non-fat milk for 2 h, the membranes were incubated with primary antibody overnight at 4 °C. Subsequently, the membranes were incubated with the corresponding secondary antibody for two hours at 37 °C, and the bands were detected using enhanced chemiluminescence (ECL) reagent under ChemiDocTM MP Imaging System (Bio-Rad). The experiment was repeated three times, and the protein band density was quantified by ImageJ software. The antibodies used in this study are listed in Additional file 1: Table S1.

### Immunofluorescence (IF)

Retinal cells were fixed with 4% paraformaldehyde for 30 min. After blocking with 5% goat serum and 0.5% Triton X-100 for 2 h, the cells were incubated with primary antibody overnight at 4 °C. Then, the cells were incubated with the corresponding secondary antibody for 2 h at room temperature. Signals were visualized with a fluorescence microscope (Thunder, Leica) after staining with DAPI.

### Enzyme-linked immunosorbent assay (ELISA)

The microglial culture medium was collected and centrifuged at 4000 rpm for 5 min, and then the supernatant was stored at -80 °C. The concentration of senescence-associated secretory phenotype (SASP)-related molecules in the supernatant was determined by an ELISA kit according to the manufacturer's instructions (NeoBioscience Technology Co. Shenzhen, China).

### Mitochondrial membrane potential

The mitochondrial membrane potential of the cells was measured using a JC-1 assay kit (MCE, Shanghai, China) for Flow Cytometry (FACSCalibur cytometer, BD) according to the manufacturer's protocol. Mitochondrial potentials were analyzed with FlowJo software as previously described [5].

### Phagocytosis assays

Fluorescent beads (diameter 0.7 µm, CellMeter, United States) are pre-incubated in fetal bovine serum at 37 °C for 1 h. The microglia were seeded into 24-well plates and treated in different groups. The cells were incubated with media containing fluorescent beads at 37 °C for 2 h. The cells were then immobilized in 4% paraformaldehyde for 20 min. The microglia were incubated overnight with rabbit anti-Iba1 antibody at 4 ℃ and then incubated with fluorescent secondary antibody. The efficiency of phagocytosis is calculated based on the weighted average of the phagocytic beads for each cell: phagocytosis efficiency (%) = (1 × X1 + 2 × X2 + 3 × X3 + 4 × X4 + 5 × X5 + 6 × X6)/ (total number of cells) × 100%, where Xn represents the number of cells containing n beads.

### Qualification of live and dead cells

The viability of RGC was determined with the LIVE/DEAD Viability Assay Kit (Invitrogen, Carlsbad, CA), which can be used to distinguish between live and dead cells. Briefly, the medium was aspirated, and the cells were incubated for 30 min in a staining solution containing 2 mM calcein-AM and 4 mM EthD-1 in PBS. After incubation, the cells were washed three times with PBS, and images were captured with a fluorescence microscope. This experiment was carried out in triplicate. Cell mortality is expressed as the percentage of dead cells relative to the total number of cells (i.e., viable plus dead cells).

### Statistical analysis

Each data point represents a cell culture triplicate of a representative experiment that was repeated at least three times. All statistical analyses were performed using GraphPad Prism 7.0 software (GraphPad, La Jolla, CA). Analysis of variance and Student's t-test was used for comparisons between groups. *P* < 0.05 was considered statistically significant.

## Results

### ATP-P2X_7_R activation induced retinal microglia senescence

The clinical characteristics of POAG and ARC patients are presented in Table 1. Compared to the < 65 old group, ATP concentration in AH slightly increased in the > 70 old group (*P* < 0.001) and the POAG group (*P* < 0.001).

**Table1:** Characteristics and comparison between primary glaucoma and control patients

	Case	Age (Y)	IOP (mmHg)	C/D ratio	MD (dB)	RNFL (μm)
Young	12	56.33 ± 5.58	13.00 ± 1.86	0.38 ± 0.06	− 7.12 ± 3.80	94.75 ± 6.66
Old	12	80.33 ± 4.75	13.25 ± 2.10	0.40 ± 0.05	− 8.72 ± 2.44	92.67 ± 7.75
Young glaucoma	12	54.23 ± 3.32	38.07 ± 6.31	0.68 ± 0.05	− 15.21 ± 9.28	67.83 ± 16.3
Old glaucoma	12	78.17 ± 3.33	37.17 ± 8.65	0.70 ± 0.06	− 16.55 ± 8.59	69.42 ± 13.6
*P1*			< 0.001	< 0.001	< 0.001	< 0.001
*P2*			< 0.001	< 0.001	< 0.001	< 0.001

Case: eye recruited into this study; *IOP* intraocular pressure, *C/D ratio* the cup-to-disk ratio

*MD* mean defect, *RNFL* retinal nerve fiber layer average thickness

*P1*: young vs young glaucoma; *P2*: old vs old glaucoma

**Fig. 1:**
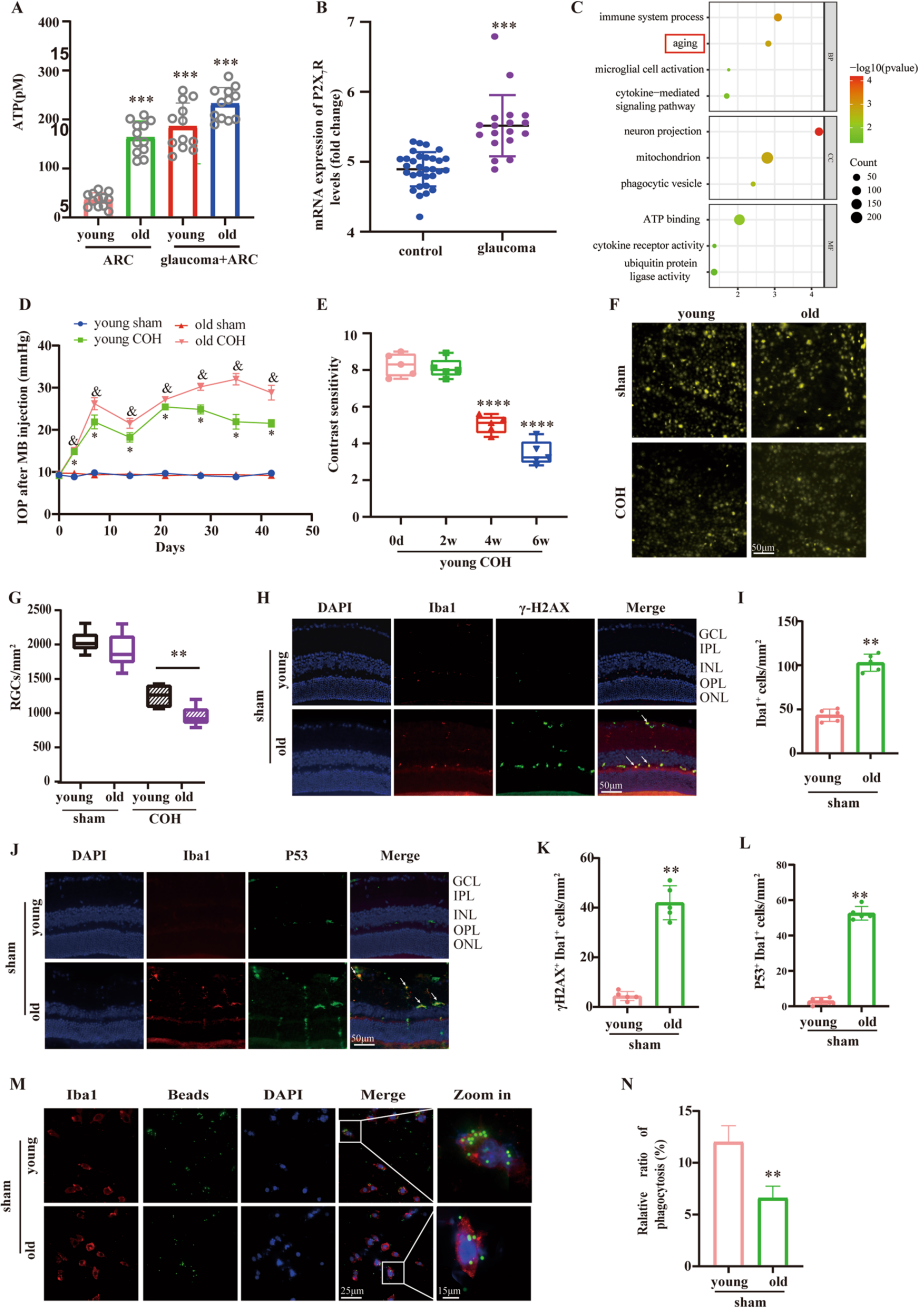
ATP-P2X_7_R activation is an important mechanism to induce retinal microglia senescence. **A** ATP concentrations in the aqueous humor in different groups. ***P < 0.001 vs. Young ARC group. **B** The expression of P2X_7_R in retina was analyzed from GSE26299 dataset. ***P < 0.001 vs. control group. **C** Functional enrichment indicated differential expressed genes between high and low P2X_7_R expression group. **D** Measurement of IOP in COH model mice. *P < 0.05 vs. Young 0 d, ^&^*P* < 0.05 vs. Old 0 d. **E** Contrast sensitivity in the COH model of young mices. ****P < 0.0001 vs. 0 d. **F**, **G** FG was injected into the superior bilateral colliculi at 7 days before COH injury injection. Fluorescence microscopy analysis of flat-mounted retinas and FG-labeled cells was performed at 6 weeks after COH injury. The remaining fluorochrome-labeled cells were quantified using image analysis and expressed as the mean number of cells 6 SEM (n = 5 per group, **P < 0.01 vs. Young COH). Scale bar: 50 μm. **H–L** Representative photomicrographs of Iba1, γ-H2AX and P53 immunofluorescence staining in the retinal extract from mice. **P < 0.01 vs. young sham. Scale bar: 50 μm. **M**, **N** The phagcytosis of microglia from adult mice retina. **P < 0.01 vs. young sham. Scale bar: 50 μm. *GCL* ganglion cell layer, *IPL* inner plexiform layer, *INL* inner core layer, *OPL* outer plexiform layer, *ONL* outer nuclear layer

To verify whether glaucoma tissues exhibited an aging phenotype, common differential geness in gene expression chip (GSE26299 datasets) from Gene Expression Omnibus (GEO) database were investigated by collecting and calculating from GEO using R language. As shown in Fig. 1B, P2X_7_R expression was upregulated in mice retina with glaucoma (control, 4.894 ± 0.2457, glaucoma, 5.516 ± 0.4371; *P* < 0.001). Subsequently, mice were divided into the high and low P2X_7_R expression groups. The result of GO enrichment analysis showed that P2X_7_R was mainly related to aging, immune system, and cytokine-mediated signaling pathways (Fig. 1C).

As shown in Fig. 1D, the mouse COH model was successfully established. The IOP increased significantly on the 3rd day after operation (*P* < 0.05), peaked at the 1st week (*P* < 0.05). We then injected microbeads again on the 14th day, and a high IOP was maintained until 6 weeks. As shown in Fig. 1E, the contrast sensitivity was decreased at 4 and 6 weeks in the COH model of young mice (*P* < 0.0001), and the RGC survival rate of the COH model of old mice was significantly lower than that of the COH model of young mice (Young COH, 1253 ± 157.5, Old COH, 850.2 ± 90.98; *P* = 0.0033) (Fig. 1F, G). In addition, the co-localization of the microglia-specific marker Iba1 and the age-related markers γ-H2AX (*P* < 0.01) and P53 (*P* < 0.01) in the retina of aged mice was increased, respectively (Fig. 1H–K). The phagocytosis of retinal microglia in aging mice was also found to be weakened (*P* < 0.01) (Fig. 1M, N). To confirm the role of P2X_7_R in microglial senescence, we use the P2X_7_R antagonist A438079 in COH model of old mice and observe the survival rate of RGC. After establishing COH model for a period of time, we found a reduction in RGC mortality (COH, 1865 ± 93.74; COH + A438079, 2245 ± 115; *P* = 0.0055) (Additional file 3: Fig. S2). These results suggested that inhibition of P2X_7_R expression reversed microglia senescence and improved RGC death. These results suggest that ATP-P2X_7_R activation may aggravate RGC damage through promoting microglia senescence in the COH model of old mice.

### BzATP-P2X_7_R activation inhibited mitophagy by inducing microglial senescence

**Fig. 2:**
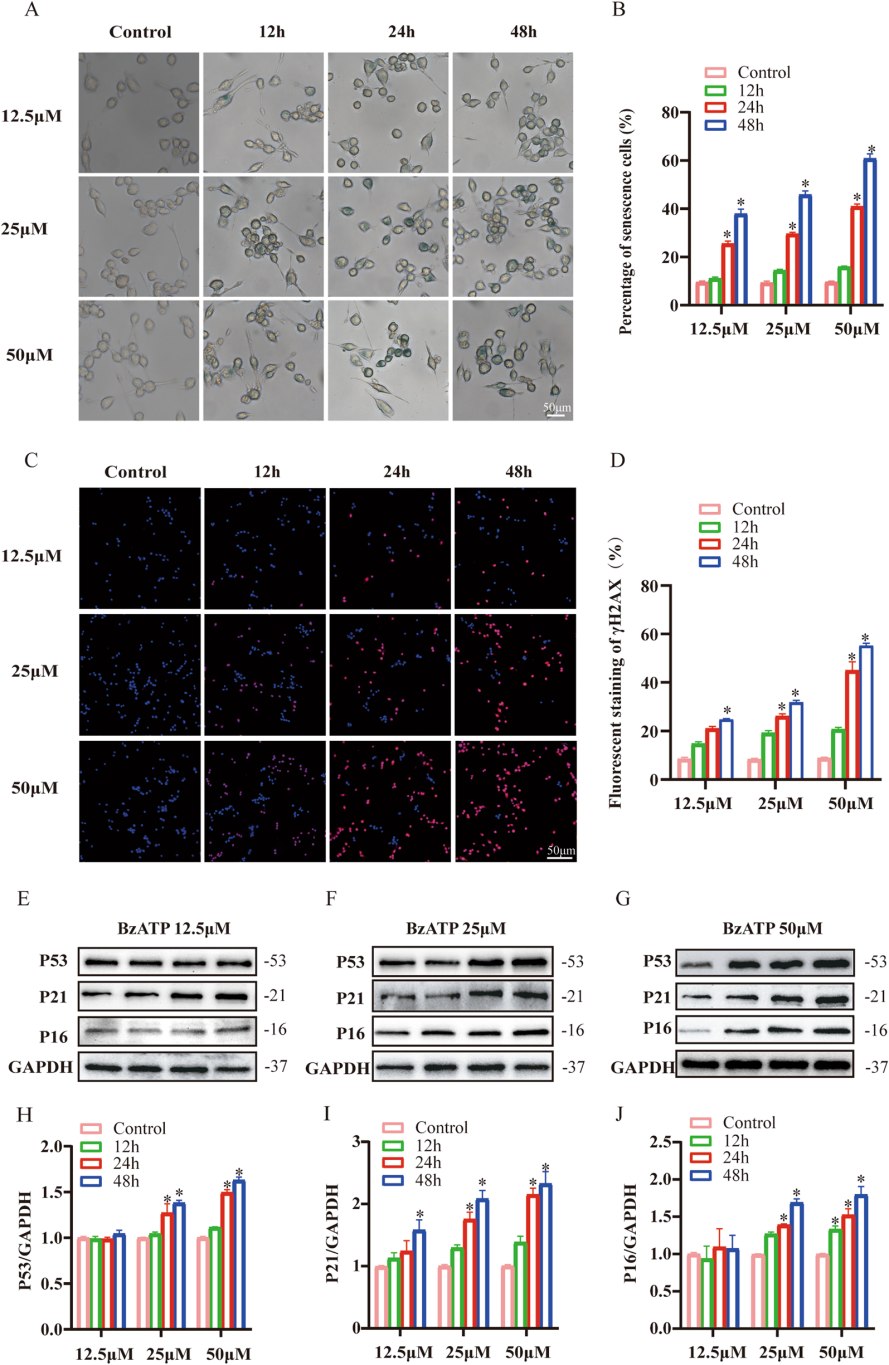
Effects of BzATP stimulation on microglial senescence in time- and dose-dependent manner. **A** The senescence of microglia was detected by SA-β-Gal. Scale bar: 50 μm. **B** Percentage of β-gal stained cells. *P < 0.05 vs. Control group. **C** γ-H2AX in microglia was detected by immunofluorescence assay. Scale bar: 50 μm. **D** Percentage of γ-H2AX stained cells. *P < 0.05 vs. Control group. **E–G** Western blot analysis of age-related markers under different concentrations of BzATP treatment. **H**–**J** Expression of **E**–**G** protein was evaluated by ImageJ. Protein expression levels were normalized according to GAPDH expression levels. Data represent the mean ± SD of three independent experiments. *P < 0.05 vs. Control group

To determine whether BzATP mediates microglial senescence and alters mitochondrial function during aging, microglia were treated with different concentrations of BzATP at different times. Elevated SA-β-Gal accumulation was observed in BV2 cells via a time and dose-dependent manner (*P* < 0.05) (Fig. 2A, B). DNA damage and age-related marker (p53, p16 and p21) are considered as hallmarks for senescence [6, 7]. As shown in Fig. 2C, D, the fluorescence intensity of γ-H2AX was elevated in a time and dose-dependent manner (*P* < 0.05). In line with γ-H2AX, the protein levels of age-related markers were significantly augmented (*P* < 0.05) (Fig. 2E–J). Similar phenomena were also detected in BzATP (50 μM) treated primary retinal microglia (*P* < 0.0001) (Additional file 4: Fig.S3), which confirmed that BzATP treatment might aggravate microglial senescence. Furthermore, we found that phagocytosis was decreased in BzATP-stimulated microglia (*P* < 0.05) (Additional file 5: Fig.S4).

**Fig. 3:**
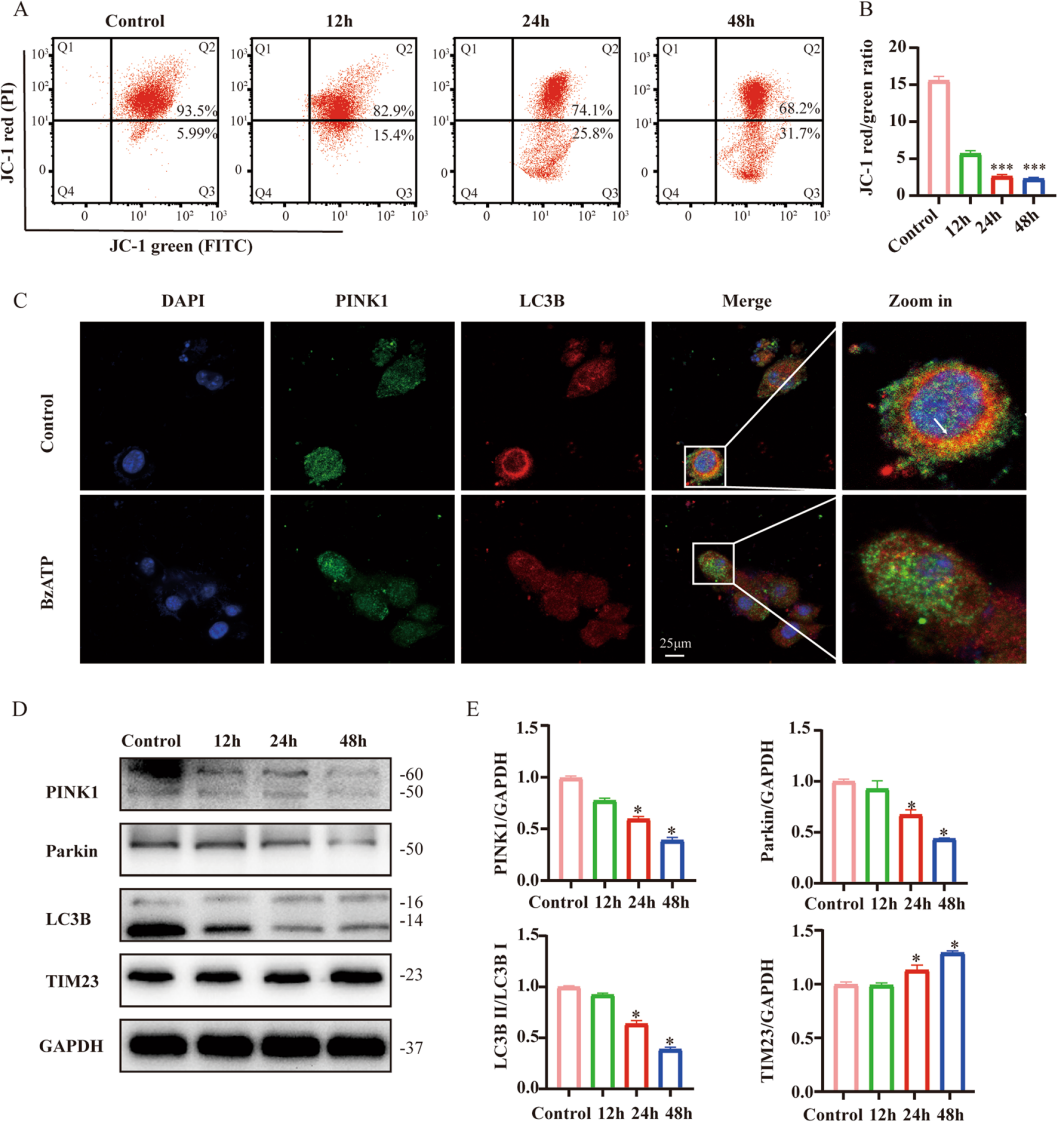
BzATP-P2X_7_R pathways specific activation of microglia mitophagy. Microglia were stimulated with 50 μM BzATP for 0-48 h. **A**, **B** Mitochondrial membrane potential was determined by JC-1 staining. The red fluorescence of the JC-1 probe indicates healthy mitochondria, while the green fluorescence of the JC-1 probe indicates damaged mitochondrial potential. ***P < 0.001 vs. Control group. **C** Representative images of PINK1 and LC3B immunofluorescence staining in control and BzATP-treated group. Scale bar: 25 μm. **D** Western blot analysis was performed to assess mitophagy-related protein levels after BzATP stimulation. **E** The expression of (D) protein was evaluated by ImageJ. Data represent the mean ± SD of three independent experiments. *P < 0.05 vs. Control group

Studies have found that senescent cells typically exhibit mitochondrial dysfunction and reduced mitophagy [8]. Thus, we speculated that BzATP might disrupt mitochondrial function. We monitored the mitochondrial membrane potential in BV2 cells after BzATP treatment. The mitochondrial membrane potential was significantly decreased in BzATP-treated cells (*P* < 0.001) (Fig. 3A, B). As reported, PINK1/Parkin pathway is of great importance for autophagic clearance of dysfunctional mitochondria [9]. By immunofluorescence staining, we found that BzATP treatment reduced the co-localization of PINK1 with LC3B (Fig. 3C). Additionally, immunoblot analysis showed decreased expression of PINK1 (*P* < 0.05) and Parkin (*P* < 0.05) in a time-dependent manner (Fig. 3D, E). Meanwhile, BzATP inhibited the conversion of LC3I to LC3II (*P* < 0.05), indicating the decreased expression of PINK1 induced by BzATP correlates with impaired mitophagy. We also found that translocase of the inner mitochondrial membrane 23 (TIM23) (*P* < 0.05) was increased, suggesting that mitochondrial clearance through mitophagy was reduced.

**Fig. 4:**
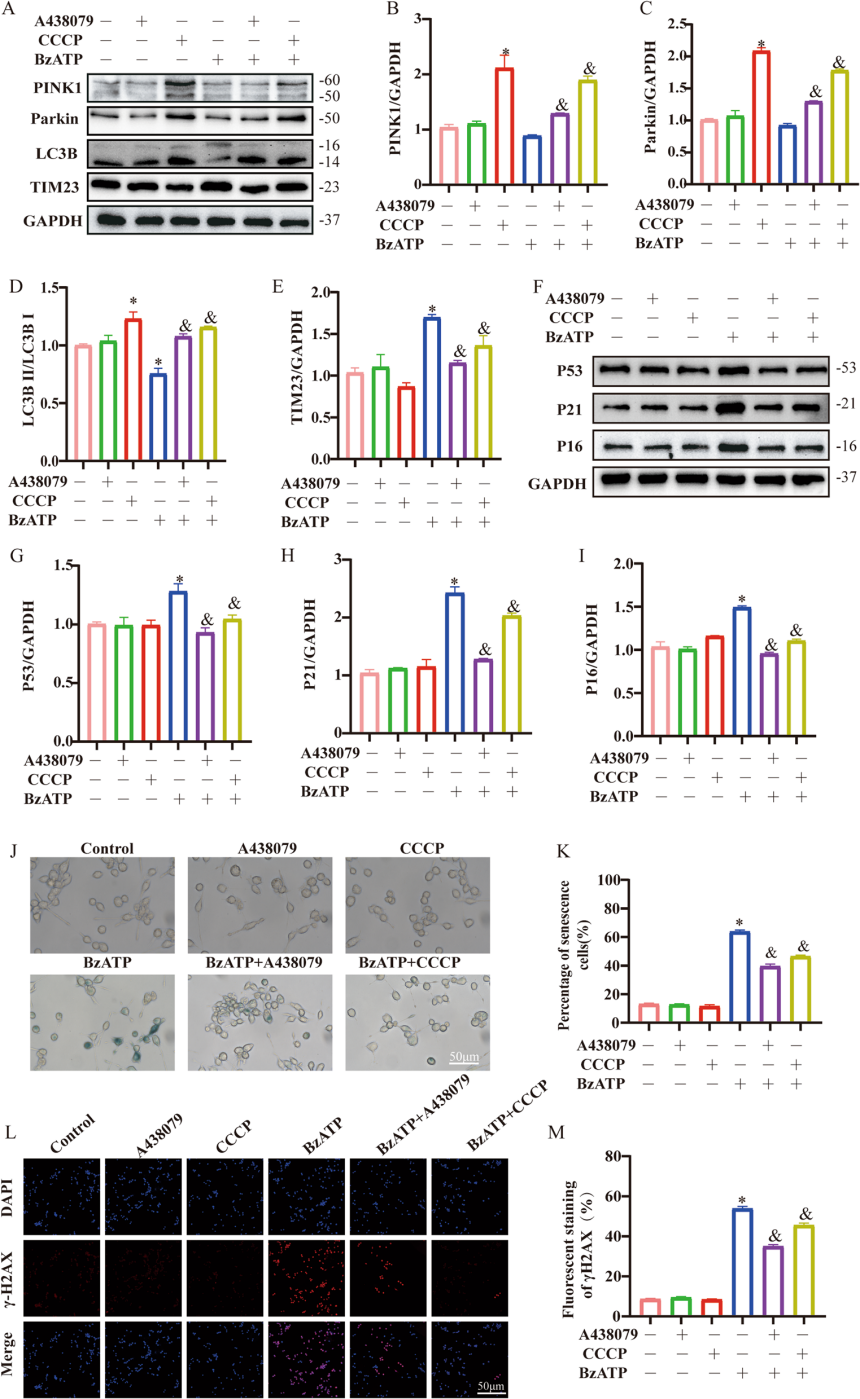
Activation of Mitophagy reverses microglial senescence. Microglia were incubated with BzATP (50 μM) combined with CCCP (5 μM) and A438079 (50 μM) treatment, respectively. **A** The protein expressions of PINK1, Parkin, LC3B and TIM23 were quantified, and GAPDH was used as the loading control. **B**–**E** Expression of **A** protein was evaluated by ImageJ. *P < 0.05 vs. Control group, *&P* < 0.05 vs. BzATP group. **F** Western blotting analysis of P53, P21 and P16 protein expression. GAPDH was used as the load control. **G**–**I** Expression of **F** protein was evaluated by ImageJ. *P < 0.05 vs. Control group, ^&^*P* < 0.05 vs. BzATP group. **J** SA-β-Gal was used to detect the senescence of microglia. **K** Percentage of β-gal stained cells. Scale bar: 50 μm. **L** γ-H2AX in microglia was detected by immunofluorescence assay. **M** Percentage of γ-H2AX stained cells. Scale bar: 50 μm

Senescent cells typically exhibit mitochondrial dysfunction and reduced mitophagy [9]. To further determine the association between mitophagy and cellular senescence, we employed A438079 (P2X_7_R inhibitor, 50 μM) and CCCP (mitophagy agonist) in BzATP-induced microglial aging. Immunoblot results revealed that CCCP restored the decreased PINK1 (*P* < 0.05), Parkin (*P* < 0.05) and LC3B (*P* < 0.05) levels, whereas reversed the elevated TIM23 levels in BzATP-treated cells (Fig. 4A–E). Compared with BzATP alone, the addition of CCCP decreased the expression of age-related markers (*P* < 0.05) (Fig. 4F–I). This was concurred by SA-β-Gal and γ-H2AX fluorescence intensity, which revealed that CCCP-activated mitophagy could attenuate BzATP-induced microglial senescence (*P* < 0.05) (Fig. 4J–M).

### Overexpression of PINK1 ameliorated BzATP-induced microglial senescence

**Fig. 5:**
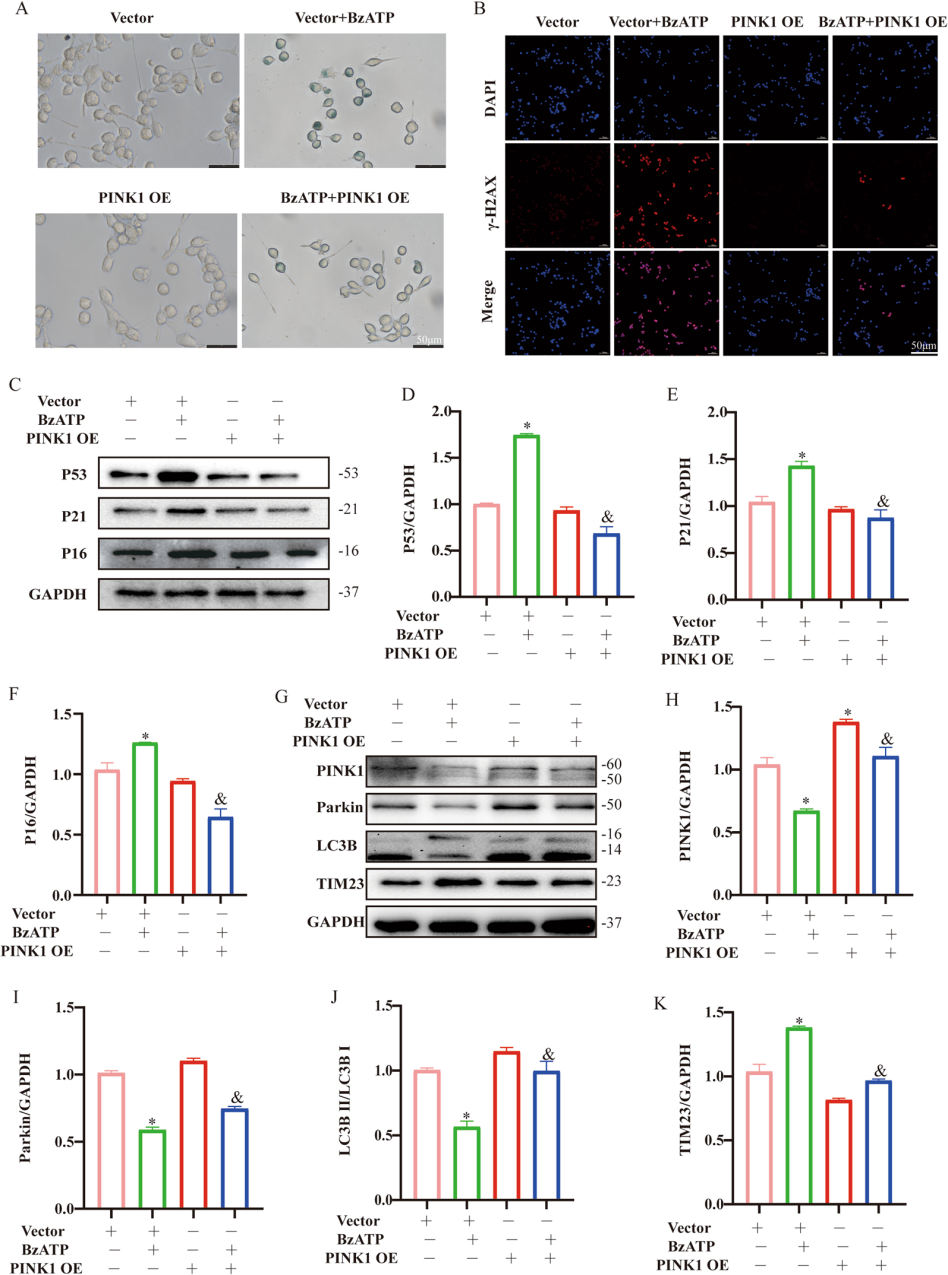
Effects of overexpression of PINK1 on mitophagy and senescence in microglia. Microglia treated with PINK1 OE and/or BzATP (50 μM, 12 h). **A** The senescence of microglia was detected by SA-β-Gal. Scale bar: 50 μm. **B** γ-H2AX in microglia was detected by immunofluorescence assay. Scale bar: 50 μm. **C** Western blotting was used to detect the protein expressions of P53, P21 and P16 after PINK1 overexpression in microglia, and GAPDH was used as the loading control. **D**–**F** Expression of **C** protein was evaluated by ImageJ, mean ± SD of three independent experiments. *P < 0.05 vs. Vector group, *&P* < 0.05 vs. BzATP + Vector group. **G** The expression of PINK1, Parkin, LC3B and TIM23 proteins was analyzed by Western blotting. GAPDH was used as the load control. **H–K** Expression of **G** protein was evaluated by ImageJ, mean ± SD of three independent experiments. *P < 0.05 vs. Vector group, ^&^*P* < 0.05 vs. BzATP + Vector group

Given that downregulation of PINK1 expression reduces mitophagy, which is closely related to microglial aging [10], we hypothesized that PINK1 could delay the aging of microglia. Numerous studies have shown that PINK1 deficiency develops aging-related diseases [11]. To confirm the role of PINK1-mediated mitophagy in BzATP-treated microglia, we overexpressed the PINK1 gene in microglia and assessed the levels of related indicators. As shown in Fig. 5A, B, SA-β-Gal (*P* < 0.05) and γ-H2AX (*P* < 0.05) staining showed that ectopic expression of PINK1 inhibited BzATP-induced cell senescence. In addition, overexpression of PINK1 decreased the expression of age-related markers (*P* < 0.05) (Fig. 5C–F). Moreover, overexpression of PINK1 alleviated BZATP-induced increased Parkin (*P* < 0.05) and LC3B (*P* < 0.05) levels, whereas upregulated the decreased TIM23 level (*P* < 0.05) (Fig. 5G–K). These results indicate that overexpression of PINK1 can activate mitophagy and reverse BzATP-induced microglial senescence.

### Overexpression of PINK1 in microglia protected RGC from glutamic acid-induced excitotoxicity

**Fig. 6:**
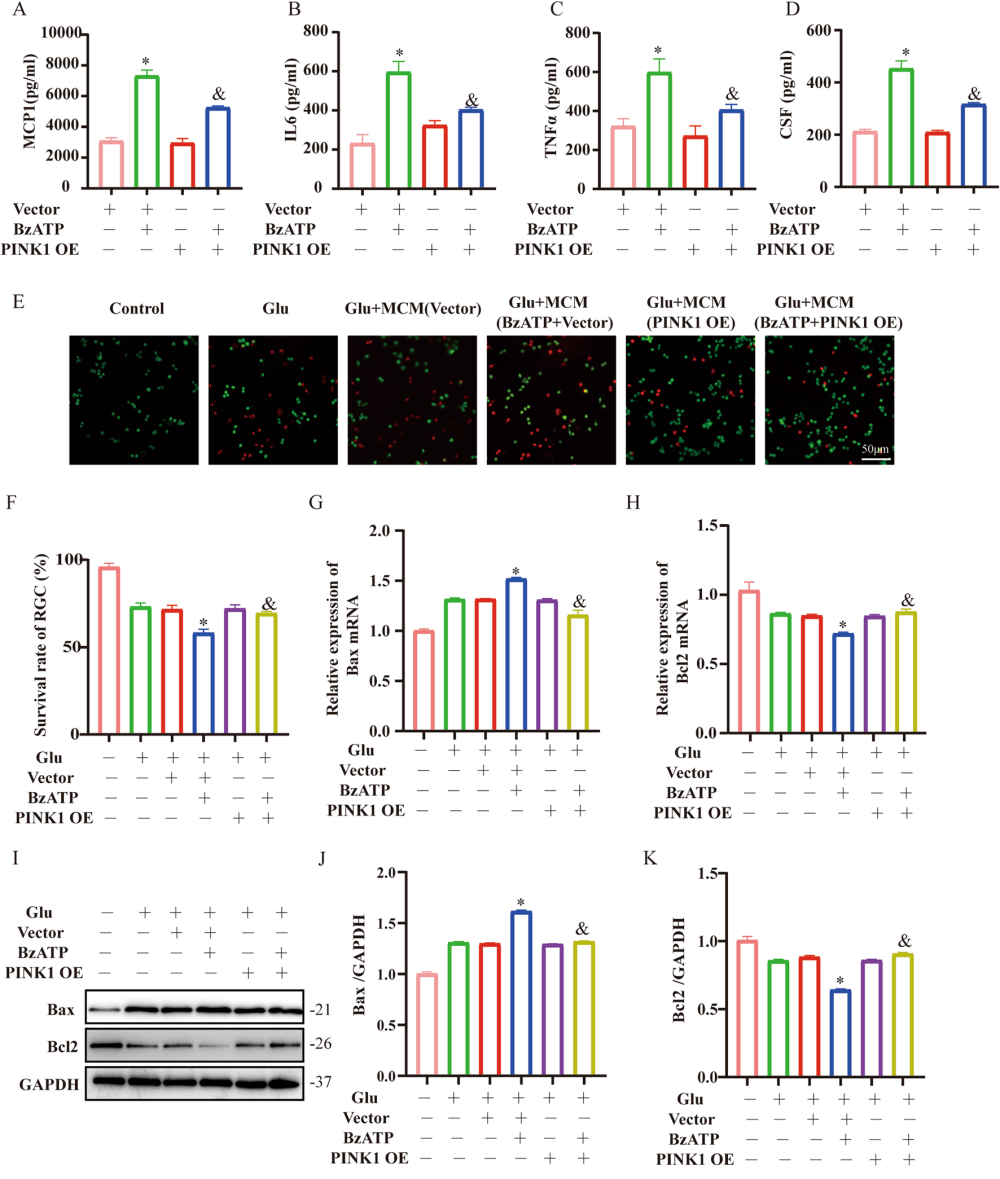
Effect of microglia overexpression of PINK1 on survival rate of RGC. Microglia treated with PINK1 OE and/or BzATP (50 μM, 12 h). **A**–**D** Secretion of SASP by microglia under different treatments. The bar graph shows the changes in extracellular concentrations of MCP1, Il-6, TNF-α and GSF under different treatments. Mean ± SD of three independent experiments. *P < 0.05 vs. Vector group, ^&^*P* < 0.05 vs. BzATP + Vector group. **E** Representative images of live (green) and dead (red) RGC after co-cultured with microglial supernatants treated with PINK1 OE and/or BzATP. Scale bar: 50 μm. **F** The bar graph summarizes the average ratio of live/dead cells under different conditions. *P < 0.05 vs. Glu, *&P* < 0.05 vs. Glu + MCM (BzATP + Vector). **G**, **H** The bar chart shows the changes in Bax and Bcl2 mRNA under different treatments. **I**–**K** The bar chart shows the changes in Bax and Bcl2 protein under different treatments. Data represent the mean ± SD of three independent experiments. *P < 0.05 vs. Vector group, ^&^*P* < 0.05 vs. BzATP + Vector group

It has been shown that senescent cells secrete increased SASP, causing changes in the surrounding microenvironment, leading to apoptosis and systemic dysfunction  [12]. Therefore, we hypothesize that enhanced mitophagy can reduce the induction of SASP and protect RGC from damage. In vitro studies demonstrated that the treatment with BzATP significantly augmented the secretion of SASP-related factors [i.e., MCP1 (Vector, 3081.23 ± 216.2, Vector + BzATP, 7391.01 ± 374.2; *P* < 0.05); TNFα (Vector, 321.7 ± 38.37, Vector + BzATP, 598.33 ± 68.6; *P* < 0.05); CSF (Vector, 207.2 ± 10.34, Vector + BzATP, 462.87 ± 22.82; *P* < 0.05) and IL-6 (Vector, 231.3 ± 45.15, Vector + BzATP, 595.7 ± 54.52; *P* < 0.05)], while these effects were blunted by PINK1 overexpression (*P* < 0.05) (Fig. 6A–D). In addition, we further demonstrated that aging microglia aggravate RGC damage in Glu using a co-culture system. Glutamic acid (Glu) was used to simulate primary RGCs in vitro, and the survival rate of RGC was determined by LIVE/DEAD assay kit. Compared to the Glu group, the RGC survival rate (*P* < 0.05) and the expression level of Bcl2 mRNA (*P* < 0.05) were significantly declined in Glu + MCM (BzATP + Vector) group, while RGC survival rate and Bcl2 mRNA expression (*P* < 0.05) were significantly increased in Glu + MCM (BzATP + PINK1 OE) group (Fig. 6E–K). Taken together, microglial senescent may accelerate RGC damage by releasing SASP and weakening phagocytosis, and enhanced microglia mitophagy may have a protective effect on RGC in the background of COH.

### Transplantation of BMCs from young mice effectively ameliorates RGC damage in the COH model of old mice

**Fig. 7:**
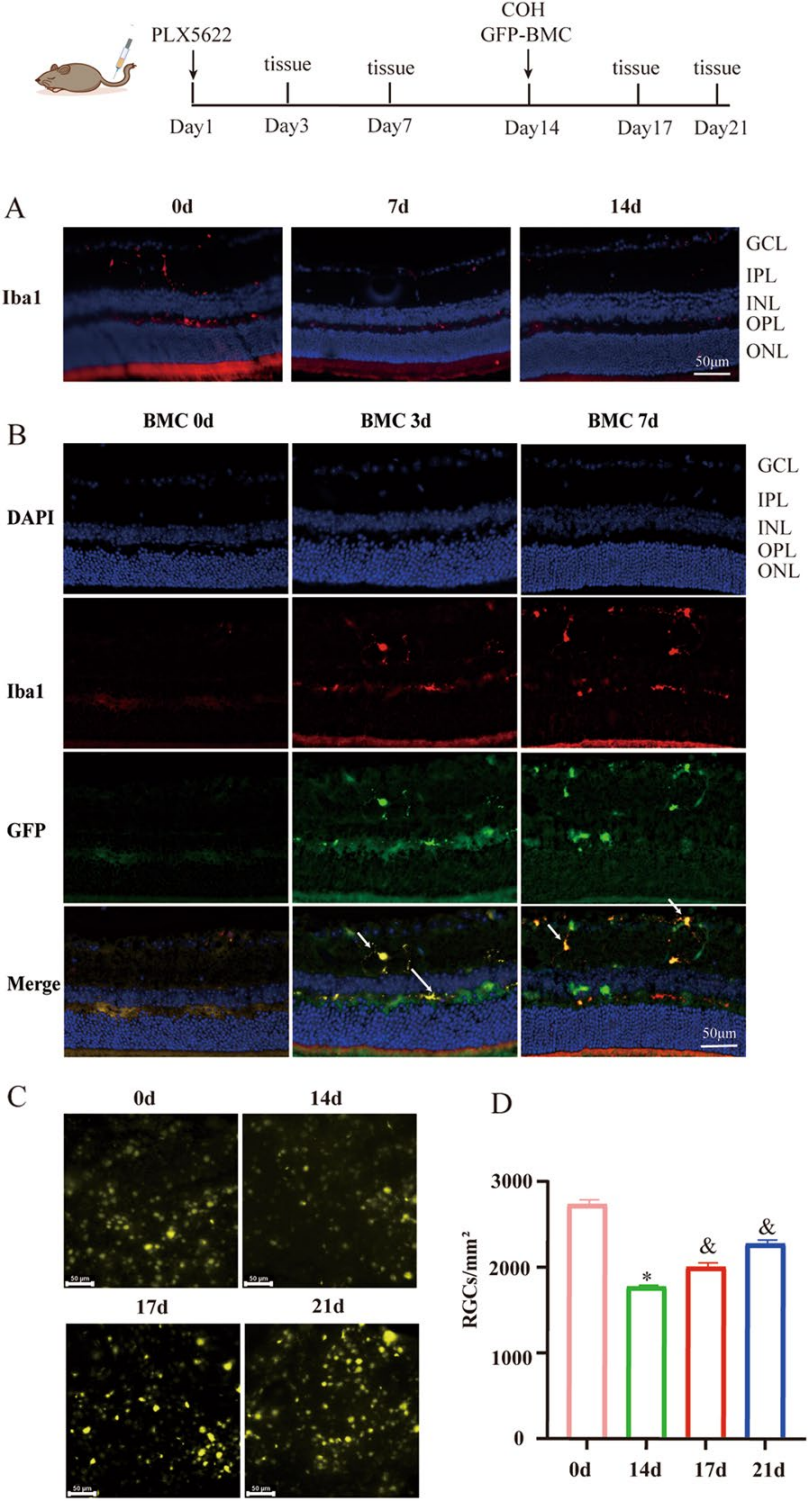
Transplantation of bone marrow cells from young mice effectively ameliorated RGC injury in COH model of aged mice. After 2 weeks of feeding on a diet formulated with 1200 ppm plx5622, microbeads were injected into the anterior chamber, and BMCs was injected into the tail vein. **A** Expression of Iba1 in retinal microglia. Scale bar: 50 μm. **B** Staining of BMCs retinas with glial labeled anti-IBA1 antibody (red) revealed three distinct labeled cell populations (red, green, and yellow appearance). Anti-iba1 antibody marks retinal microglia (red). GFP represents differentiated cells after the migration of bone marrow cells into the retina (green). Yellow round microglia indicate the differentiation of bone marrow cells into microglia (arrows). Scale bar: 50 μm. **C**, **D** FG-labeled RGC were analyzed by fluorescence microscopy after bone marrow transplantation. Scale bar: 50 μm. Each dataset is expressed as mean ± SD. *P < 0.05 vs. 0 d, ^&^*P* < 0.05 vs. 14d. *GCL* ganglion cell layer, *IPL* inner plexiform layer, *INL* inner core layer, *OPL* outer plexiform layer, *ONL* outer nuclear layer

Since loss-of-function of microglia may fail to protect RGC by altering the retinal microenvironment [13], we evaluated whether bone marrow transplantation that contains macroglia from young mice ameliorated RGC damage induced by the COH model in aged mice. Consistent with previous reports [14], we found that retinal microglia cells were almost completely lost at 2 weeks after PLX5622 treatment, with less than 1% of Iba1-positive cells surviving (Fig. 7A). Iba1-positive microglia showed strong activation at 3 and 6 days after BMCs transplantation (Fig. 7B). Up to 5 weeks, a distinct green cell population was visible in the retina (Additional file 6: Fig. S5). FG labeling of RGC showed that BMCs transplantation prevented retinal RGC injury in COH model of old mice (*P* < 0.05) (Fig. 7C, D).

## Discussion

The pathogenesis of glaucoma involves many factors, among which the senescence of microglia plays an important role [15]. Meanwhile, age is a major risk factor for glaucoma and has been included in glaucoma risk calculations [19]. However, the exact mechanism underlying the relationship between aging and glaucoma development has not been fully elucidated. In this study, we found that long-term low concentration of ATP in AH from the elderly and POAG patients induced P2X_7_R activation, leading to retinal microglial senescence. Mechanistically, pathological activation of ATP-P2X_7_R inhibited the PINK1-mediated mitophagy pathway. Additionally, senescent microglia accelerated RGC damage in COH by increasing SASP secretion. Importantly, replacement of senescent retinal microglia in COH model of old mice by injection of BMCs from young mice effectively ameliorated RGC injury.

Microglia are the primary innate immune cells in retina. Microglia-mediated immune and inflammatory response were considered to be the possible mechanism for the development of glaucoma [20]. Normal microglia play an essential role in the maintenance of normal neuronal function, primarily through phagocytosis of debris and the initiation of complex molecular cascades to fight infections. Senescent microglia with reduced ramifications and cytoplasmic swelling can be seen in pathological states and the healthy brains of elderly individuals [21]. Microglial senescence is thought to be involved in the release of toxic reactive oxygen species, inflammatory cytokines and decreased phagocytosis, thereby causes neuronal dysfunction [22]. Studies have shown that microglia surface prostaglandin receptor EP2 can be activated through GSK-3β pathway, which promotes mitochondrial glycogen synthesis and leads to macrophage/microglia senescence. EP2 inhibitor can block the aging of microglia and improve mitochondrial energy metabolism, which can make the inflammatory aging of the whole body and brain return to the young state [23]. Previous studies have shown that P2X_7_, P2X_4_ and P2Y_12_ receptors were involved in microglia activation, proliferation and migration, which contribute to RGC damage in COH model [24, 25]. Studies have detected mild increase of ATP concentration in diseased retinal tissue, AH in patients with acute and chronic angle-closure glaucoma, and retina in aging mice with chronic spontaneous ocular hypertension [26–29]. Meanwhile, high concentration of BzATP induces the activation of microglia, leading to inflammatory response [30]. In our study, the extracellular ATP concentration in the retina of elderly individuals and glaucoma patients was in a state of long-term mild increase. However, no relevant reports have been reported on the functional changes of microglia stimulated by long-term low concentration of BzATP. Our study found that 12 h after BzATP stimulation of microglia cells, the activity of SA-β-Gal increased, the expression of age-related marker proteins significantly increased, and the phagocytosis function decreased, confirming the occurrence of senescence.

Many studies have shown that increased cellular senescence may be due to increased age-dependent mitochondrial damage or decreased age-dependent mitophagy and decreased capacity to clear dysfunctional mitochondria [31]. There is evidence that both immune aging and low-grade chronic inflammation, as a feature of aging, are associated with impaired autophagic flux in microglia [32]. Our findings suggested that CCCP-mediated activation of mitophagy protects RGCs by reducing microglial senescence. Numerous studies have identified PINK1 as a key component in the mitochondrial homeostasis pathway, which is closely related to aging due to its role in scavenging damaged mitochondria through mitophagy [33]. Our study showed that BzATP stimulation of microglia reduced PINK1 expression and accelerated microglial senescence, while PINK1 overexpression activated mitophagy and inhibited microglial senescence induced by P2X_7_R activation, suggesting that induction of PINK1 expression is a potential strategy for the treatment of glaucoma.

The mechanism of immune damage has attracted much attention in glaucoma research in recent years. In the early stage of glaucoma, the secretion of TGF-β2 by microglia down-regulates the degenerative reaction in the optic nerve and maintains the homeostasis of the microenvironment as macrophage phagocytic cell fragments, thus playing a beneficial function in glaucomatous optic nerve injury [34]. However, our experiments showed that aging microglia cells respond slowly to pathological stimulation, which was manifested as the weakened ability to phagocytic damaged and dead RGC. Importantly, the continuous secretion of SASP such as MCP1, TNF-α, CSF and IL6 accelerated the death of RGC, leading to the aggravation of glaucoma.

As reported, transplantation of bone marrow stem cells, injection of macrophages into the bloodstream and injection of microglia directly into injured sites can treat age-related neurodegenerative diseases [35, 36]. Recruitment of bone marrow-derived cells may require several signaling steps: mobilization of monocytes from the bone marrow and blood; transport through the blood–retina barrier, including adhesion and lysis; attraction to sites of injury within the retina; and conversion to active macrophages [37]. We found strong microglial activation and normal microglial morphology 3 and 7 days after BMCs transplantation, and showed that BMCs transplantation alleviated RGC injury in the COH model of old mice without altering visual function. We suspect that newborn microglia have better migration ability, which serve as phagocytosis of damaged RGC; On the one hand, it reduces the secretion of SASP and achieves the effect of protecting RGC. Therefore, we further hypothesized that young microglia can be transplanted into COH model of old mice to replace senescent microglia or in combination with other therapies to prevent RGC damage.

**Fig. 8:**
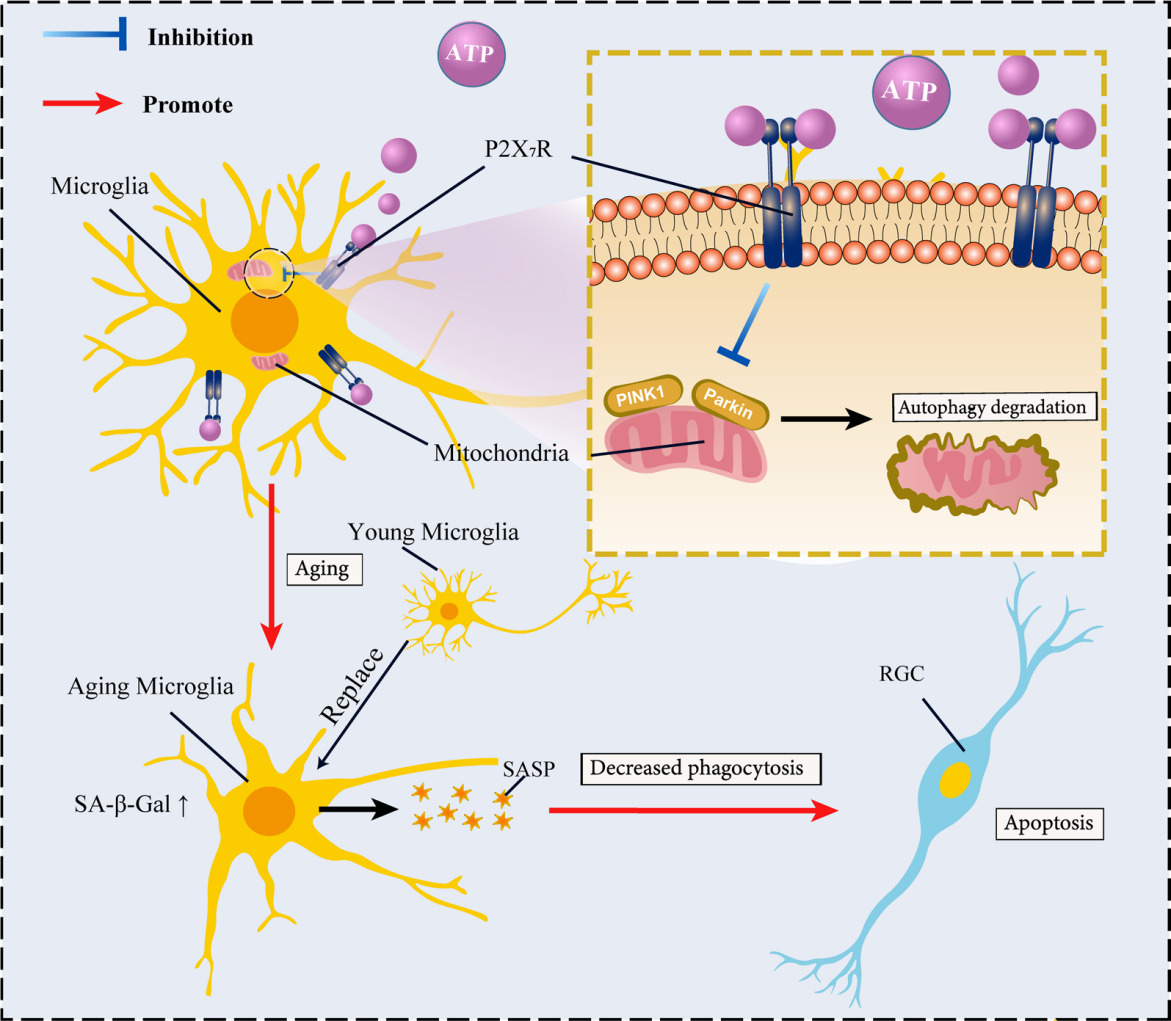
Mechanism diagram of ATP-P2X_7_R pathway mediates microglial senescence to accelerate RGC damage in COH

This study is the first to describe a novel mechanism by which microglial senescence is induced by ATP-P2X_7_R in glaucoma progression (Fig. 8). Through in vivo and in vitro approaches, we demonstrated that ATP-P2X_7_R aggravated retinal microglia senescence via suppressing PINK1-mediated mitophagy pathway, thereby accelerated RGC death in COH model. More importantly, replacement of senescent microglia in COH model of old mice via tail vein injection of BMCs from young mice can alleviate RGC death. Specific inhibition of microglia ATP-P2X_7_R or immunotherapy may be the basic strategies to delay the development of glaucoma.

## Supplementary Information

**Additional file 1: Table S1.** Antibodies information.

**Additional file 2. Figure S1.** Primary cell culture identification. **A** Representative image of Iba1-labeled mouse BV2 cell line. Scale bar: 100μm. **B** Representative images of Iba1-labeled primary mouse retinal microglia. Scale bar: 50μm. **C** Representative images of RBPMS labeled primary RGC in mouse retina. Scale bar: 50μm.

**Additional file 3. Figure S2.** The RGC survival rate. **A**, **B** RGC survival rate after vitreous injection of A438079 in mice. n = 5 in each group. *P < 0.05 vs. control, ^&^*P* < 0.05 vs. COH. Scale bar: 50μm.

**Additional file 4. Figure S3.** BzATP-P2X_7_R specific activation promotes retina primary microglia senescence. Primary retina microglia were stimulated with 50μM BzATP for 24h. **A** SA-β-Gal was used to detect senescent cells (white arrows). Scale bar: 50 μm. **B** Percentage of β-gal stained cells. ****P < 0.0001 vs. Control group. **C** Representative images of γ-H2AX fluorescence staining after BzATP stimulation of primary microglia. Scale bar: 50 μm. **D** Percentage of γ-H2AX stained cells. *P < 0.05 vs. Control group. **E** Western blot analysis was performed for age-related markers in primary microglia. **F** Expression of **E** protein was evaluated by ImageJ. **P < 0.01 vs. Control group. (**G**–**H**) The phagocytic ability of microglia. Data represent the mean ± SD of three independent experiments. **P < 0.01 vs. Control group. Scale bar: 25 μm.

**Additional file 5. Figure S4.** The phagocytosis ability of BV2. ** A**, **B** The phagocytosis ability of BV2 was decreased after stimulation with 50μM BzATP for 24h. *P < 0.05 vs. Control group. Scale bar: 25 μm.

**Additional file 6. Figure S5.** Fluorescence detection of bone marrow cells. GFP cells were still visible in the mouse retina 5 weeks after tail vein injection. Scale bar: 100μm.

## Data Availability

Gene expression profiling in DBA/2J glaucoma from GSE26299 (https://www.ncbi.nlm.nih.gov/geo/). Other data generated in this study are included in this manuscript.

## Abbreviations

COH: Chronic ocular hypertensive
ATP: Adenosine triphosphate
CCCP: Carbonyl cyanide m-chlorophenylhydrazone
PINK1: PTEN-induced kinase 1
RGC: Retinal ganglion cells
IOP: Intraocular pressure
P2X_7_R: P2X_7_ receptor
BMCs: Bone marrow cells
ARC: Age-related cataract
ELISA: Enzyme-linked immunosorbent assay
IL6: Interleukin-6
TNFα: Tumor necrosis factor alpha
MCP1: Monocyte chemoattractant protein 1
SASP: Senescence-associated secretory phenotype
siRNA: Small interference RNA
SA-β-Gal: Senescence-associated β-galactosidase
TIM23: The inner mitochondrial membrane 23

## References

[1] Howell GR, Macalinao DG, Sousa GL, Walden M, Soto I, Kneeland SC (2011). Molecular clustering identifies complement and endothelin induction as early events in a mouse model of glaucoma. J Clin Invest.

[2] Chen H, Wei X, Cho KS, Chen G, Sappington R, Calkins DJ (2011). Optic neuropathy due to microbead-induced elevated intraocular pressure in the mouse. Invest Ophthalmol Vis Sci.

[3] Ji M, Sun Q, Zhang G, Huang Z, Zhang Y, Shen Q (2022). Microglia-derived TNF-alpha mediates Muller cell activation by activating the TNFR1-NF-kappaB pathway. Exp Eye Res.

[4] Wei M, Chen LM, Huang ZY, Zhang GW, Guan HJ, Ji M (2022). Expression profile analysis to identify potential gene changes induced by dexamethasone in the trabecular meshwork. Int J Ophthalmol.

[5] Sun J, Liu X, Shen C, Zhang W, Niu Y (2021). Adiponectin receptor agonist AdipoRon blocks skin inflamm-ageing by regulating mitochondrial dynamics. Cell Prolif.

[6] Mandal PK, Blanpain C, Rossi DJ (2011). DNA damage response in adult stem cells: pathways and consequences. Nat Rev Mol Cell Biol.

[7] Barnes PJ, Baker J, Donnelly LE (2019). Cellular senescence as a mechanism and target in chronic lung diseases. Am J Respir Crit Care Med.

[8] Cai Q, Jeong YY (2020). Mitophagy in Alzheimer's disease and other age-related neurodegenerative diseases. Cells.

[9] Iorio R, Celenza G, Petricca S (2021). Mitophagy: molecular mechanisms, new concepts on parkin activation and the emerging role of AMPK/ULK1 axis. Cells.

[10] Sliter DA, Martinez J, Hao L, Chen X, Sun N, Fischer TD (2018). Parkin and PINK1 mitigate STING-induced inflammation. Nature.

[11] Sachdeva K, Do DC, Zhang Y, Hu X, Chen J, Gao P (2019). Environmental exposures and asthma development: autophagy, mitophagy, and cellular senescence. Front Immunol.

[12] Sun Y, Wang X, Liu T, Zhu X, Pan X (2022). The multifaceted role of the SASP in atherosclerosis: from mechanisms to therapeutic opportunities. Cell Biosci.

[13] Cheng X, Gao H, Tao Z, Yin Z, Cha Z, Huang X (2023). Repopulated retinal microglia promote Muller glia reprogramming and preserve visual function in retinal degenerative mice. Theranostics..

[14] Todd L, Palazzo I, Suarez L, Liu X, Volkov L, Hoang TV (2019). Reactive microglia and IL1beta/IL-1R1-signaling mediate neuroprotection in excitotoxin-damaged mouse retina. J Neuroinflammation.

[15] Ramirez AI, Fernandez-Albarral JA, Hoz R, Lopez-Cuenca I, Salobrar-Garcia E, Rojas P (2020). Microglial changes in the early aging stage in a healthy retina and an experimental glaucoma model. Prog Brain Res.

[16] Bohlen CJ, Bennett FC, Bennett ML (2019). Isolation and culture of microglia. Curr Protoc Immunol.

[17] You MJ, Rim C, Kang YJ, Kwon MS (2021). A new method for obtaining bankable and expandable adult-like microglia in mice. J Neuroinflammation.

[18] Devarajan G, Chen M, Muckersie E, Xu H (2014). Culture and characterization of microglia from the adult murine retina. ScientificWorldJournal.

[19] Williams PA, Harder JM, Foxworth NE, Cochran KE, Philip VM, Porciatti V (2017). Vitamin B3 modulates mitochondrial vulnerability and prevents glaucoma in aged mice. Science.

[20] Wei X, Cho KS, Thee EF, Jager MJ, Chen DF (2019). Neuroinflammation and microglia in glaucoma: time for a paradigm shift. J Neurosci Res.

[21] Angelova DM, Brown DR (2019). Microglia and the aging brain: are senescent microglia the key to neurodegeneration?. J Neurochem.

[22] Ogrodnik M, Evans SA, Fielder E, Victorelli S, Kruger P, Salmonowicz H (2021). Whole-body senescent cell clearance alleviates age-related brain inflammation and cognitive impairment in mice. Aging Cell.

[23] Minhas PS, Latif-Hernandez A, McReynolds MR, Durairaj AS, Wang Q, Rubin A (2021). Restoring metabolism of myeloid cells reverses cognitive decline in ageing. Nature.

[24] Jing F, Zhang Y, Long T, He W, Qin G, Zhang D (2019). P2Y12 receptor mediates microglial activation via RhoA/ROCK pathway in the trigeminal nucleus caudalis in a mouse model of chronic migraine. J Neuroinflammation.

[25] Campagno KE, Lu W, Jassim AH, Albalawi F, Cenaj A, Tso HY (2021). Rapid morphologic changes to microglial cells and upregulation of mixed microglial activation state markers induced by P2X7 receptor stimulation and increased intraocular pressure. J Neuroinflammation.

[26] Pedata F, Dettori I, Coppi E, Melani A, Fusco I, Corradetti R (2016). Purinergic signalling in brain ischemia. Neuropharmacology.

[27] Li A, Zhang X, Zheng D, Ge J, Laties AM, Mitchell CH (2011). Sustained elevation of extracellular ATP in aqueous humor from humans with primary chronic angle-closure glaucoma. Exp Eye Res.

[28] Perez de Lara MJ, Guzman-Aranguez A, de la Villa P, Diaz-Hernandez JI, Miras-Portugal MT, Pintor J. Increased levels of extracellular ATP in glaucomatous retinas: possible role of the vesicular nucleotide transporter during the development of the pathology. Mol Vis. 2015;21:1060–70. PMC4558477 26392744

[29] Lu W, Hu H, Sevigny J, Gabelt BT, Kaufman PL, Johnson EC (2015). Rat, mouse, and primate models of chronic glaucoma show sustained elevation of extracellular ATP and altered purinergic signaling in the posterior eye. Invest Ophthalmol Vis Sci.

[30] Zhang Y, Xu Y, Sun Q, Xue S, Guan H, Ji M (2019). Activation of P2X(7)R- NLRP3 pathway in retinal microglia contribute to retinal ganglion cells death in chronic ocular hypertension (COH). Exp Eye Res.

[31] Maremanda KP, Sundar IK, Li D, Rahman I (2020). Age-dependent assessment of genes involved in cellular senescence, telomere, and mitochondrial pathways in human lung tissue of smokers, COPD, and IPF: associations with SARS-CoV-2 COVID-19 ACE2-TMPRSS2-Furin-DPP4 axis. Front Pharmacol.

[32] Yousefzadeh MJ, Flores RR, Zhu Y, Schmiechen ZC, Brooks RW, Trussoni CE (2021). An aged immune system drives senescence and ageing of solid organs. Nature.

[33] Liu T, Yang Q, Zhang X, Qin R, Shan W, Zhang H (2020). Quercetin alleviates kidney fibrosis by reducing renal tubular epithelial cell senescence through the SIRT1/PINK1/mitophagy axis. Life Sci.

[34] Yuan L, Neufeld AH (2001). Activated microglia in the human glaucomatous optic nerve head. J Neurosci Res.

[35] Xu R, Li X, Boreland AJ, Posyton A, Kwan K, Hart RP (2020). Human iPSC-derived mature microglia retain their identity and functionally integrate in the chimeric mouse brain. Nat Commun.

[36] Minhas PS, Liu L, Moon PK, Joshi AU, Dove C, Mhatre S (2019). Macrophage de novo NAD(+) synthesis specifies immune function in aging and inflammation. Nat Immunol.

[37] Joly S, Francke M, Ulbricht E, Beck S, Seeliger M, Hirrlinger P (2009). Cooperative phagocytes: resident microglia and bone marrow immigrants remove dead photoreceptors in retinal lesions. Am J Pathol.

