# Supplementary material for: ATP-P2X7R-mediated microglia senescence aggravates retinal ganglion cell injury in chronic ocular hypertension
Source: J Neuroinflammation. 2023 Jul 31;20:180. doi: 10.1186/s12974-023-02855-1 (PMC10392012; doi:10.1186/s12974-023-02855-1)
Supplement: Supplementary file 1 — Additional file 1: Table S1. Antibodies information. [file 12974_2023_2855_MOESM1_ESM.docx]

Additional file 1: Table S1: Antibodies

| Target | Product information | Diluted concentration |
| --- | --- | --- |
| P2X_7_R | Proteintech; #28207-1-AP | WB: 1:1000; IF: 1:200 |
| LC3B | Abcam; #ab48394 | WB: 1:1500; IF: 1:200 |
| γ-H2AX | Abcam; #ab81299 | IF: 1:200 |
| P53 | CST;#2524 | WB: 1:1000; IF: 1:200 |
| P21 | CST;#2947 | WB: 1:1000 |
| P16 | CST;#29271 | WB: 1:1000 |
| PINK1 | ABclonal; #A11453 | WB: 1:800 |
| Parkin | ABclonal; #A0968 | WB: 1:800 |
| TIM23 | Santa cruz#sc-514463 | WB: 1:1000 |
| RBPMS | Proteintech; #15187-1-AP | IF: 1:200 |
| Iba1 | Proteintech; #66827-1-Ig  Wako; #019-19741 | IF: 1:500 |
| GAPDH | ABclonal; #AC001 | WB: 1:6000 |
| Anti-Rabbit HRP | Jacksonimmuno Research; #111-005-003 | WB: 1:10000 |
| Anti-mouse HRP | Jacksonimmuno Research; #115-035-003 | WB: 1:10000 |
| Alexa Fluor 568  Goat anti-rabbit IgG | Invitrogen; #A11011 | IF: 1:200 |
| Alexa Fluor 568  Goat anti-mouse IgG | Invitrogen; #A11004 | IF: 1:200 |
| Alexa Fluor 488  Goat anti-rabbit IgG | Invitrogen; #A11008 | IF: 1:200 |
| Alexa Fluor 488  Goat anti-mouse IgG | Invitrogen; #A11001 | IF: 1:200 |

| 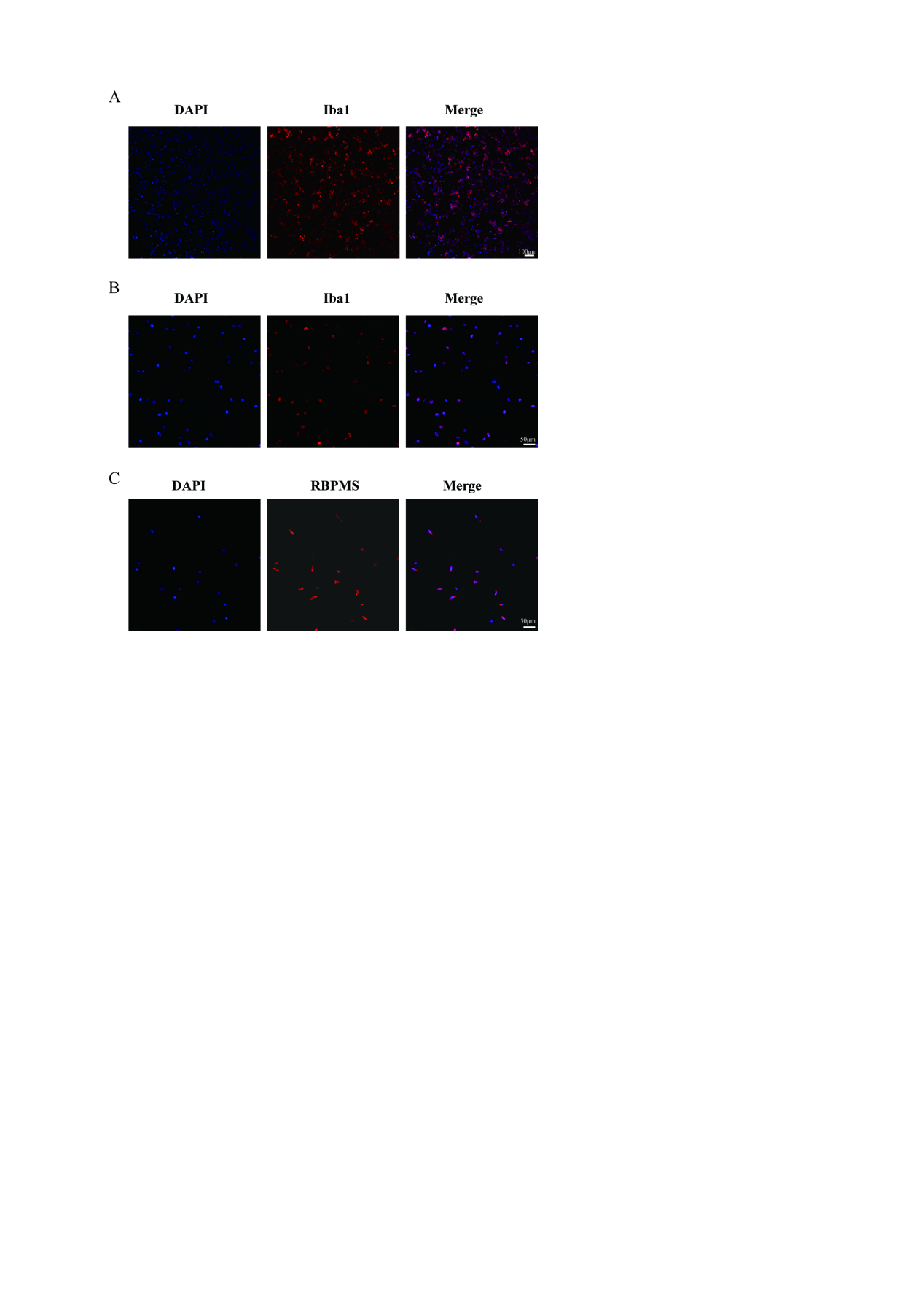 |
| --- |
| Additional file 1: **Figure S1:** Primary cell culture identification. (A). Representative image of Iba1-labeled mouse BV2 cell line. Scale bar: 100μm. (B). Representative images of Iba1-labeled primary mouse retinal microglia. Scale bar: 50μm. (C). Representative images of RBPMS labeled primary RGC in mouse retina. Scale bar: 50μm. |
| **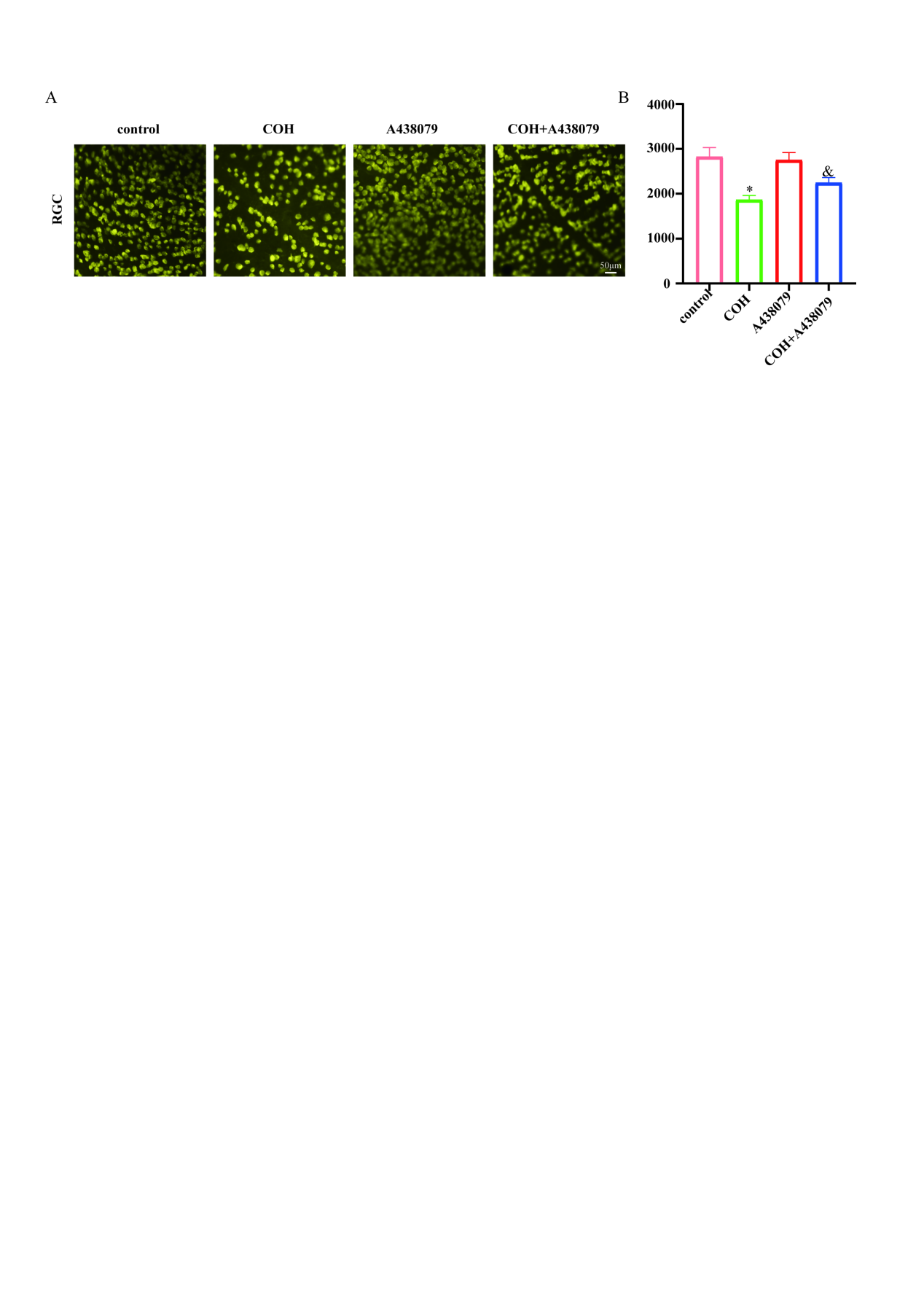** |
| Additional file 1: **figure S2:** (A-B).RGC survival rate after vitreous injection of A438079 in mice. n=5 in each group. **P* < 0.05 vs. control, *&P* < 0.05 vs. COH. Scale bar: 50μm. |
| **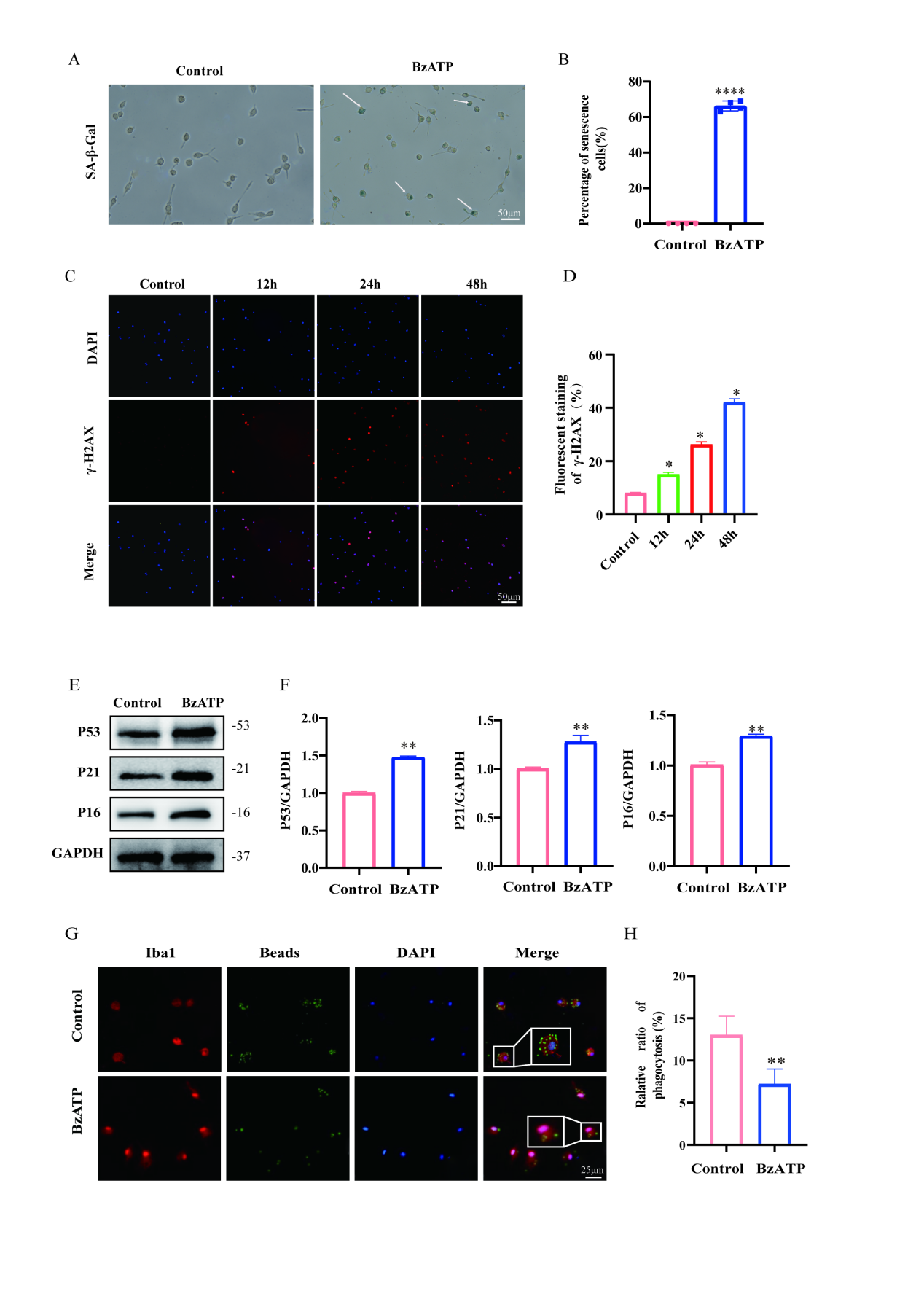** |
| Additional file 1: **Figure S3:** BzATP-P2X_7_R specific activation promotes retina primary microglia senescence. Primary retina microglia were stimulated with 50μM BzATP for 24h. (A) SA-β-Gal was used to detect senescent cells (white arrows). Scale bar: 50 μm. (B) Percentage of β-gal stained cells. **** *P* < 0.0001 vs. Control group. (C) Representative images of γ-H2AX fluorescence staining after BzATP stimulation of primary microglia. Scale bar: 50 μm. (D) Percentage of γ-H2AX stained cells. **P* < 0.05 vs. Control group. (E) Western blot analysis was performed for age-related markers in primary microglia. (F) Expression of (E) protein was evaluated by ImageJ. ***P* < 0.01 vs. Control group. (G-H) The phagocytic ability of microglia. Data represent the mean ± SD of three independent experiments. ***P* < 0.01 vs. Control group. Scale bar: 25 μm. |
| **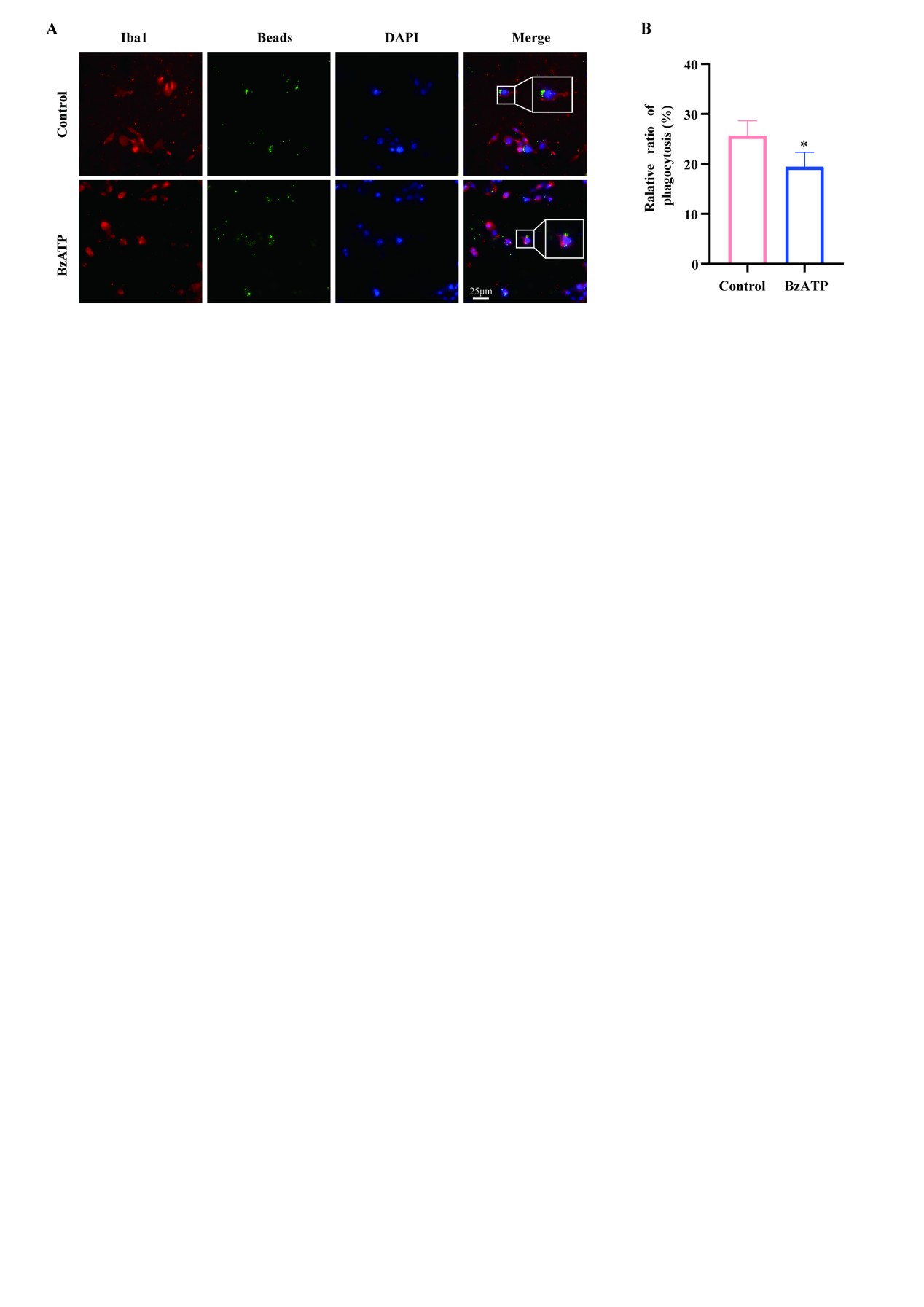** |
| Additional file 1: **Figure S4：**(A-B). The phagocytosis ability of BV2 was decreased after stimulation with 50μM BzATP for 24h. **P* < 0.05 vs. Control group. Scale bar: 25 μm. |
| 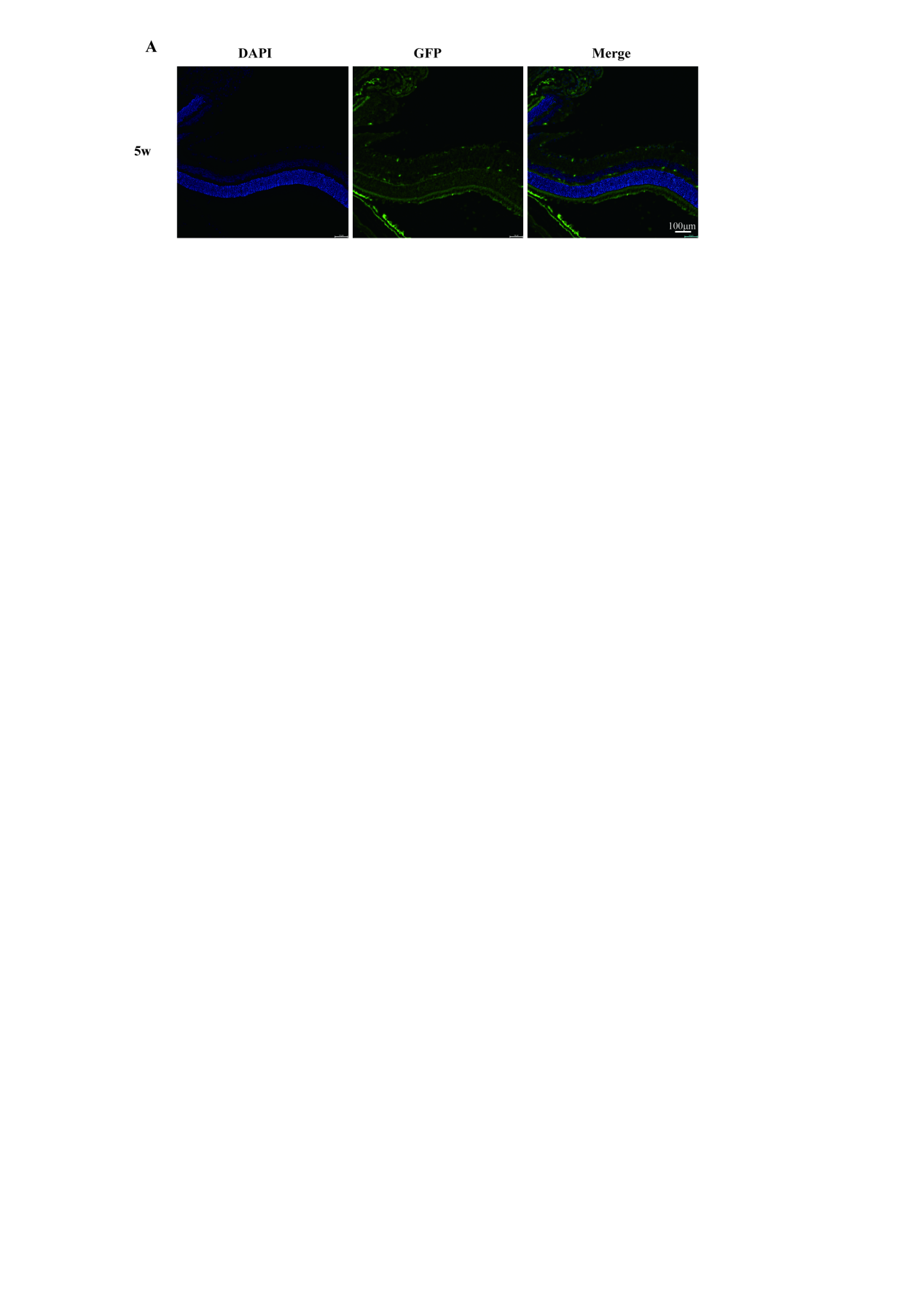 |
| Additional file 1: **figure S5:** GFP cells were still visible in the mouse retina 5 weeks after tail vein injection. Scale bar: 100μm. |
