# Supplementary figures and images for: ATP-P2X7R-mediated microglia senescence aggravates retinal ganglion cell injury in chronic ocular hypertension
Source: J Neuroinflammation. 2023 Jul 31;20:180. doi: 10.1186/s12974-023-02855-1 (PMC10392012; doi:10.1186/s12974-023-02855-1)

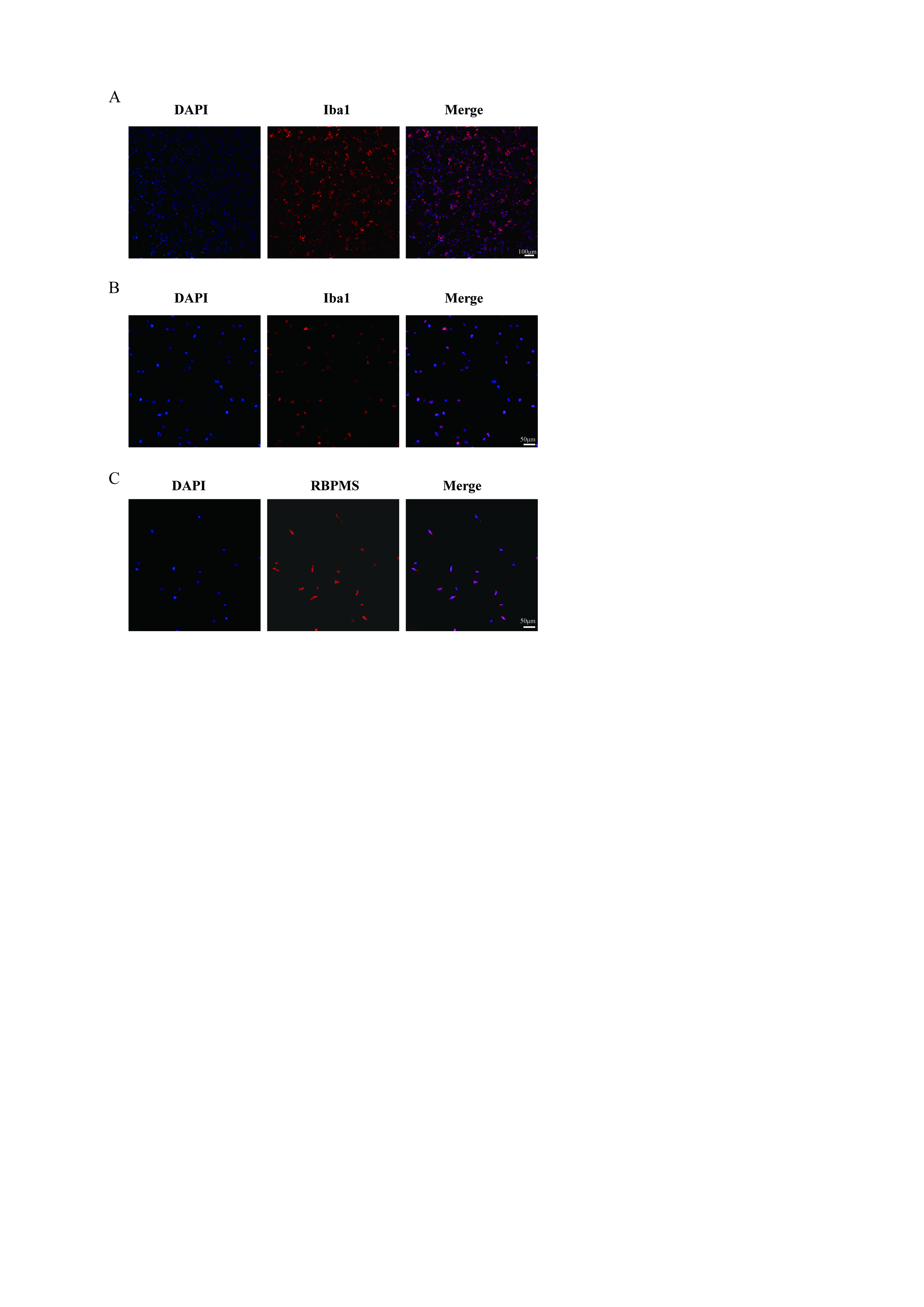

Supplement: Supplementary file 2 — Additional file 2. Figure S1. Primary cell culture identification. A Representative image of Iba1-labeled mouse BV2 cell line. Scale bar: 100μm. B Representative images of Iba1-labeled primary mouse retinal microglia. Scale bar: 50μm. C Representative images of RBPMS labeled primary RGC in mouse retina. Scale bar: 50μm. [file 12974_2023_2855_MOESM2_ESM.tif]

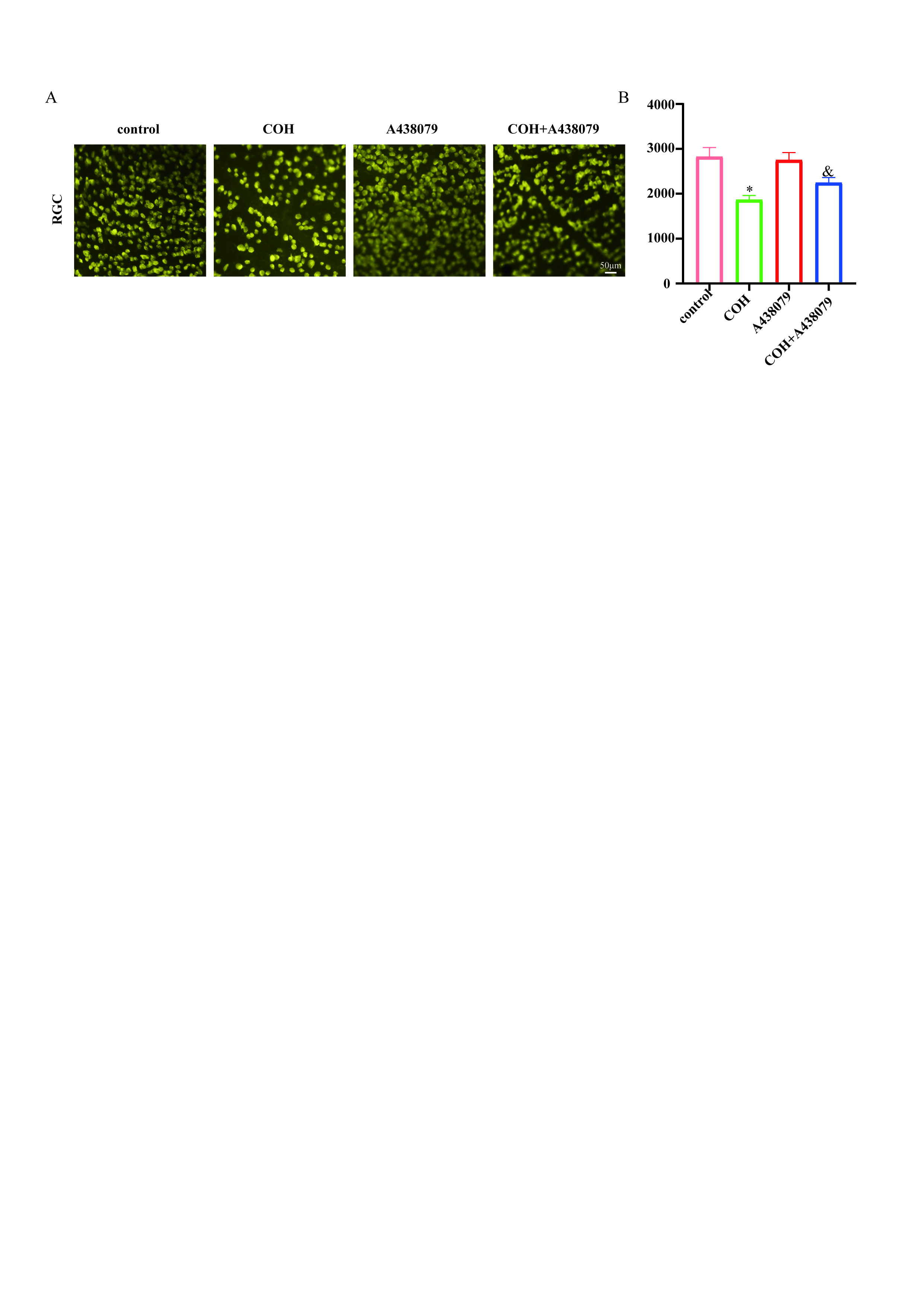

Supplement: Supplementary file 3 — Additional file 3. Figure S2. The RGC survival rate. A, B RGC survival rate after vitreous injection of A438079 in mice. n = 5 in each group. *P < 0.05 vs. control, &P < 0.05 vs. COH. Scale bar: 50μm. [file 12974_2023_2855_MOESM3_ESM.tif]

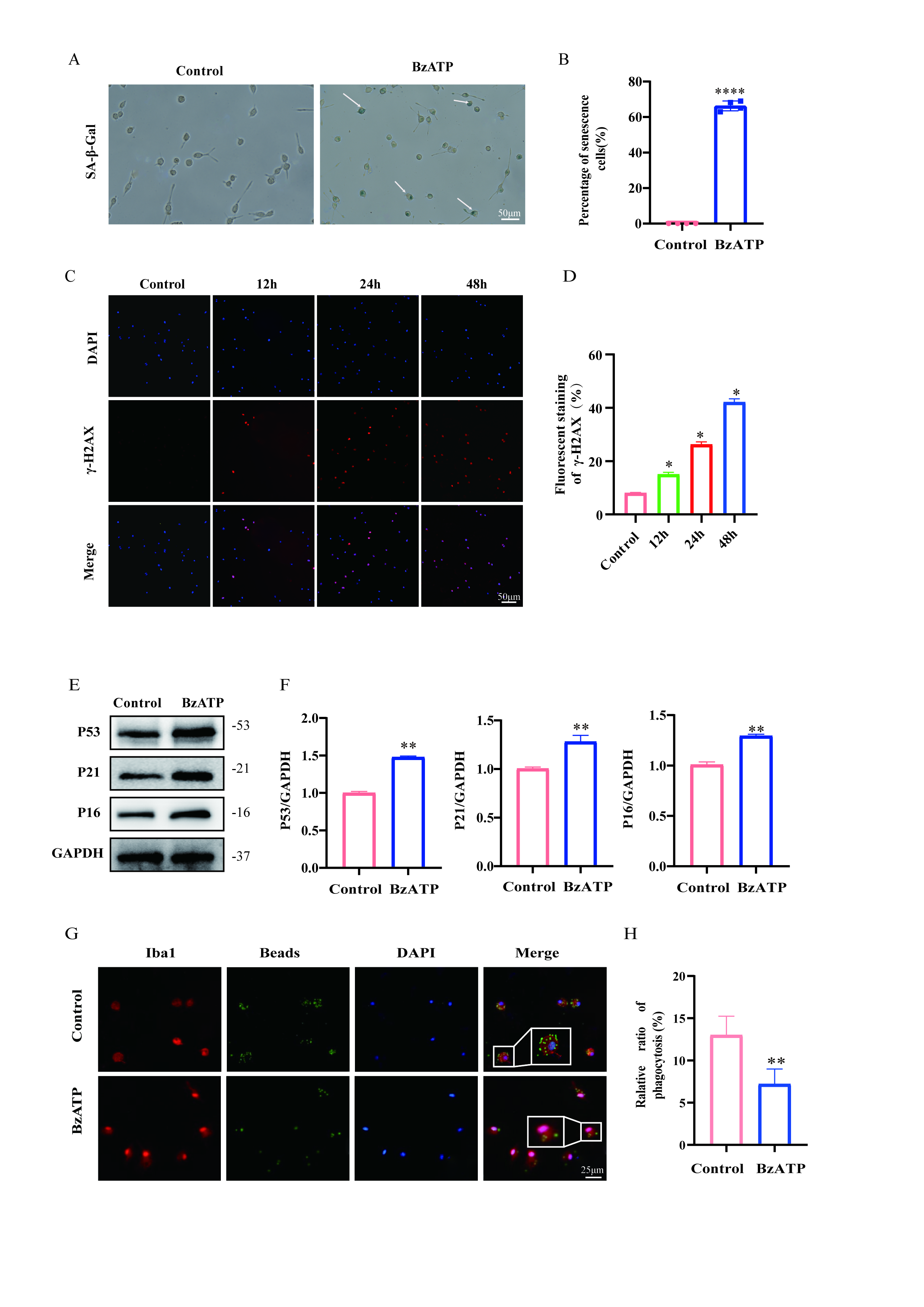

Supplement: Supplementary file 4 — Additional file 4. Figure S3. BzATP-P2X7R specific activation promotes retina primary microglia senescence. Primary retina microglia were stimulated with 50μM BzATP for 24h. A SA-β-Gal was used to detect senescent cells (white arrows). Scale bar: 50 μm. B Percentage of β-gal stained cells. ****P < 0.0001 vs. Control group. C Representative images of γ-H2AX fluorescence staining after BzATP stimulation of primary microglia. Scale bar: 50 μm. D Percentage of γ-H2AX stained cells. *P < 0.05 vs. Control group. E Western blot analysis was performed for age-related markers in primary microglia. F Expression of E protein was evaluated by ImageJ. **P < 0.01 vs. Control group. (G–H) The phagocytic ability of microglia. Data represent the mean ± SD of three independent experiments. **P < 0.01 vs. Control group. Scale bar: 25 μm. [file 12974_2023_2855_MOESM4_ESM.tif]

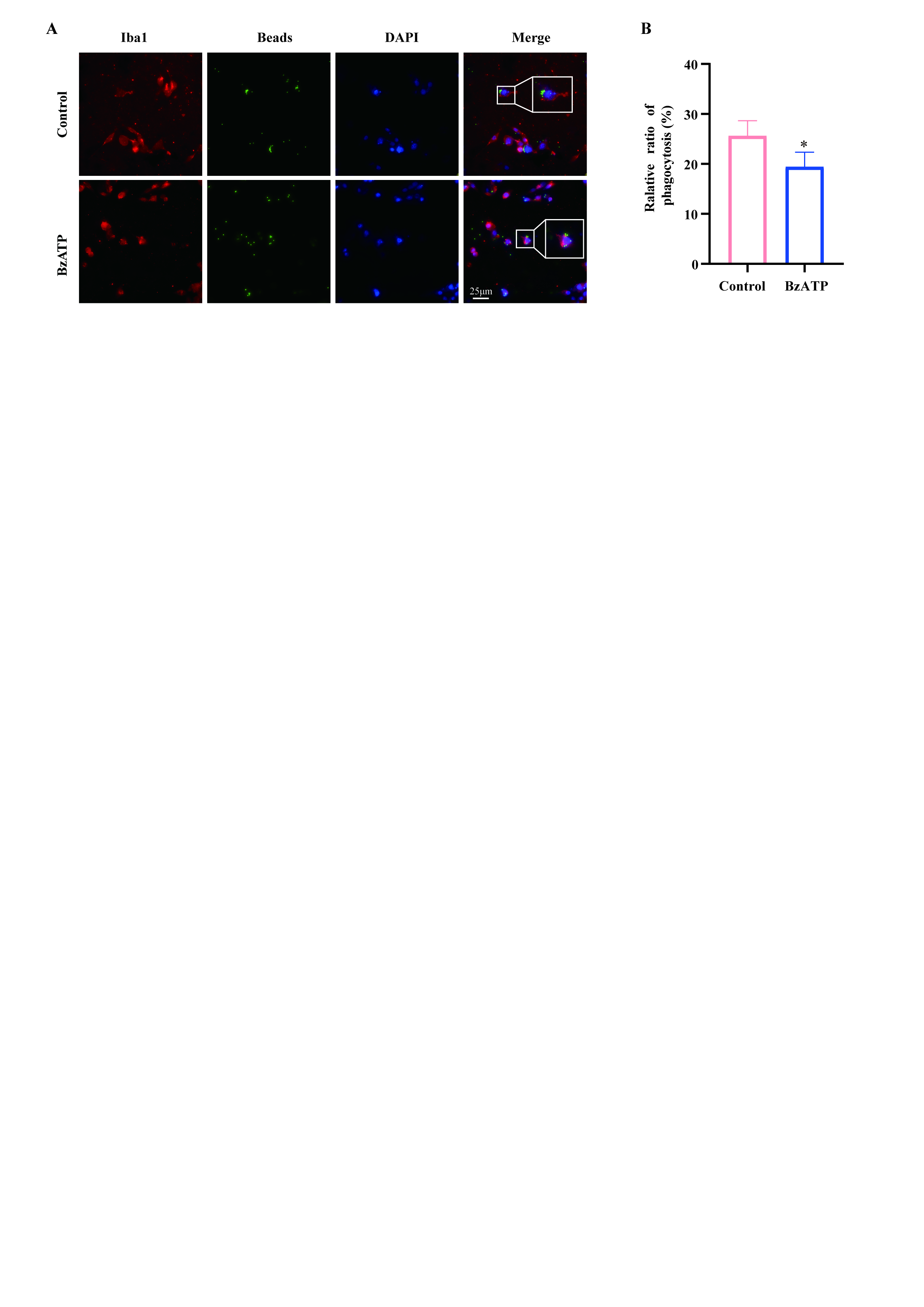

Supplement: Supplementary file 5 — Additional file 5. Figure S4. The phagocytosis ability of BV2. A, B The phagocytosis ability of BV2 was decreased after stimulation with 50μM BzATP for 24h. *P < 0.05 vs. Control group. Scale bar: 25 μm. [file 12974_2023_2855_MOESM5_ESM.tif]

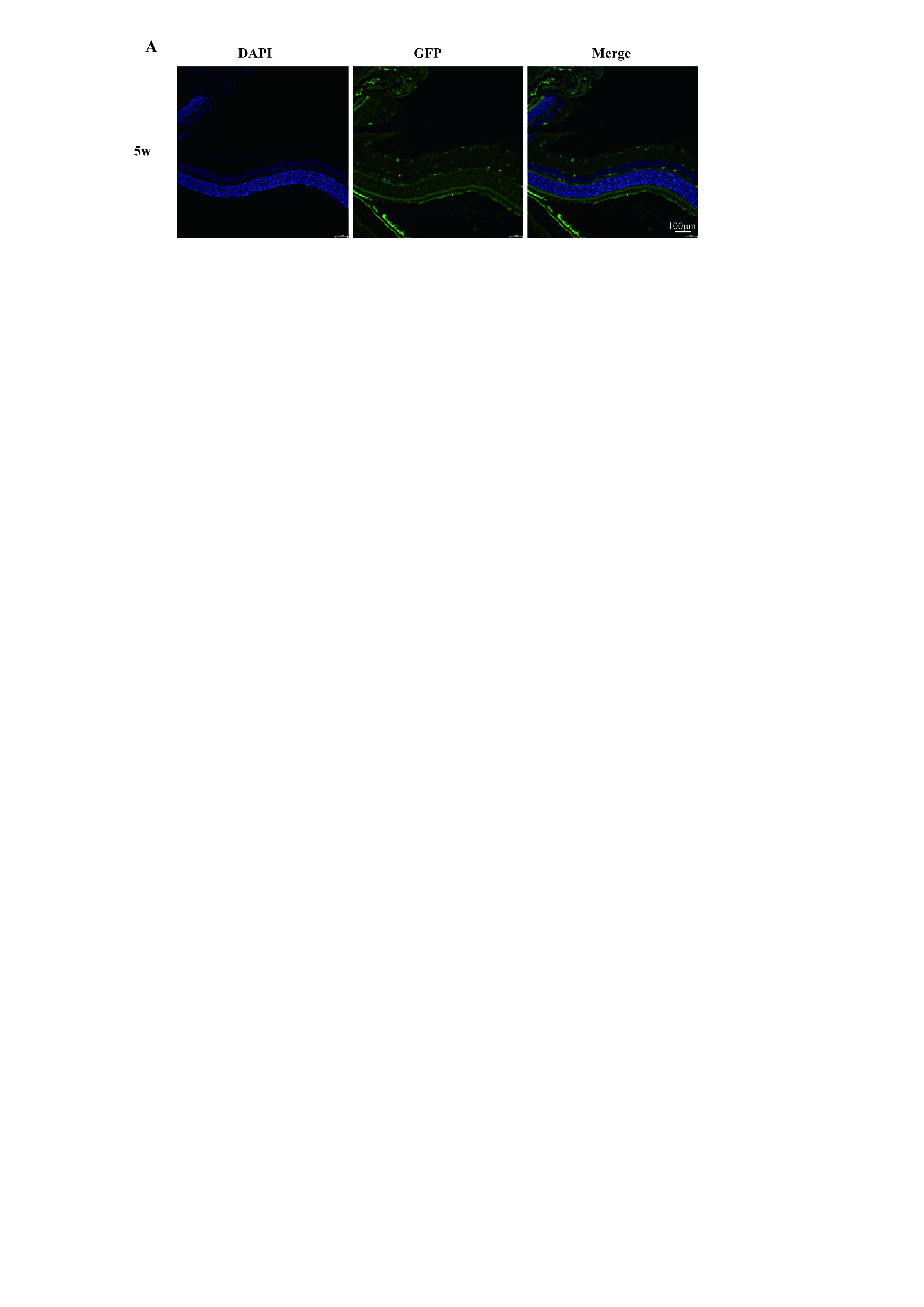

Supplement: Supplementary file 6 — Additional file 6. Figure S5. Fluorescence detection of bone marrow cells. GFP cells were still visible in the mouse retina 5 weeks after tail vein injection. Scale bar: 100μm. [file 12974_2023_2855_MOESM6_ESM.tif]
